# Forest type modulates mammalian responses to megafires

**DOI:** 10.1038/s41598-024-64460-3

**Published:** 2024-06-12

**Authors:** Marcelo Magioli, Luanne Helena Augusto Lima, Priscilla Marqui Schmidt Villela, Ricardo Sampaio, Lilian Bonjorne, Renan Lieto Alves Ribeiro, Daniel Luis Zanella Kantek, Selma Samiko Miyazaki, Thiago B. F. Semedo, Gustavo S. Libardi, Bruno H. Saranholi, Charlotte E. Eriksson, Ronaldo Gonçalves Morato, Christian Niel Berlinck

**Affiliations:** 1 Instituto Pró-Carnívoros, Atibaia, São Paulo Brazil; 2 Centro Nacional de Pesquisa e Conservação de Mamíferos Carnívoros, Instituto Chico Mendes de Conservação da Biodiversidade, Atibaia, São Paulo Brazil; 3 Laboratório de Ecologia e Conservação (LAEC), Departamento de Biologia, Faculdade de Filosofia, Ciências e Letras de Ribeirão Preto (FFCLRP), Universidade de São Paulo, Ribeirão Preto, Brazil; 4 EcoMol Consultoria, Piracicaba, São Paulo Brazil; 5 Estação Ecológica de Taiamã, Instituto Chico Mendes de Conservação da Biodiversidade, Cáceres, Mato Grosso Brazil; 6 Centro Nacional de Pesquisa e Conservação de Mamíferos Aquáticos, Instituto Chico Mendes de Conservação da Biodiversidade, Santos, São Paulo Brazil; 7 InBIO Laboratório Associado, CIBIO, Centro de Investigação em Biodiversidade e Recursos Genéticos, Universidade do Porto, Campus de Vairão, 4485-661 Vairão, Portugal; 8 BIOPOLIS Program in Genomics, Biodiversity and Land Planning, CIBIO, Campus de Vairão, 4485-661 Vairão, Portugal; 9 Departamento de Biologia, Faculdade de Ciências, Universidade do Porto, 4099-002 Porto, Portugal; 10 Facultad de Ciencias Exactas, Físicas y Naturales, Universidad Nacional de Córdoba, Córdoba, Argentina; 11 Departamento de Genética e Evolução, Universidade Federal de São Carlos, São Carlos, Brazil; 12 Department of Fisheries and Wildlife, Oregon State University, Corvallis, OR 97331 USA; 13 Departamento de Conservação e Uso Sustentável da Biodiversidade, Secretaria Nacional de Biodiversidade, Floresta e Direito dos Animais, Ministério do Meio Ambiente e Mudança Clima, Brasília, Brazil

**Keywords:** Pantanal, Brazil, Wildfire, Occupancy models, Camera trap, Environmental DNA, Relative abundance, Community ecology, Conservation biology, Fire ecology, Wetlands ecology

## Abstract

Although considered an evolutionary force responsible for shaping ecosystems and biodiversity, fires’ natural cycle is being altered by human activities, increasing the odds of destructive megafire events. Here, we show that forest type modulates the responses of terrestrial mammals, from species to assemblage level, to a catastrophic megafire in the Brazilian Pantanal. We unraveled that mammalian richness was higher 1 year after fire passage compared to a pre-fire condition, which can be attributed to habitat modification caused by wildfires, attracting herbivores and open-area tolerant species. We observed changes in assemblage composition between burned/unburned sites, but no difference in mammalian richness or relative abundance. However, by partitioning the effects of burned area proportion per forest type (monospecific vs. polyspecific), we detected differential responses of mammals at several levels of organization, with pronounced declines in species richness and relative abundance in monospecific forests. Eighty-six percent of the species presented moderate to strong negative effects on their relative abundance, with an overall strong negative effect for the entire assemblage. Wildfires are predicted to be more frequent with climate and land use change, and if events analogous to Pantanal-2020 become recurrent, they might trigger regional beta diversity change, benefitting open-area tolerant species.

## Introduction

Although fire is perceived as a destructive agent, more recently, it has been considered an evolutionary force^1^, shaping ecosystems, biodiversity, and human behavior^2^. Nonetheless, Earth's warming and drying climate, combined with changes in land use and biodiversity composition induced by human activities, are altering the natural global fire activity^3^, increasing the frequency of devastating wildfire events worldwide^4^. Wildfire impacts vary with their frequency and intensity^5^, the type of vegetation^6^, the target organisms, and their associated life history traits^7,8^. Different combinations of these aspects can drive animal mortality across vast areas, exerting strong selective pressure on wildlife populations^9^, mediated by direct (burning and intoxication), indirect (starvation, dehydration, lack of shelter), and evolutionary effects (fire history)^10^.

Even in fire-prone ecosystems, if wildfire occurrence or behavior changes suddenly, for example, more ignitions, extreme fire weather, high fuel loads, or uncharacteristically low fuel moisture (or combinations thereof), a mismatch between the historical association of risk with a particular fire cue by wildlife can be expected, with misinterpretation of danger and maladaptive decisions^1^. In 2020, after a historic drought^11^, the Pantanal of Brazil, one of the largest freshwater wetlands in the world^12^, faced the biggest wildfires ever recorded, resulting in severe ecosystem destruction and massive biodiversity loss^13^. According to Menezes et al.^14^, between 2012 and 2017, anthropogenic fires accounted for 95% of the wildfires in Pantanal, mostly occurring in the dry season, when lightning strikes (the only natural cause of wildfires in Brazil) are less expected and wildlife is not adapted to (see^15^).

The estimated burned area in Pantanal during the megafire of 2020 surpassed 4.5 million hectares^16^, resulting in the death of 17 million vertebrates^13^, and negative impacts on at least 65 million animals due to habitat loss and resource scarcity (food, water, shelter) caused by environmental changes^17^. One of the main aspects of this event was burning areas not subject to or less affected by wildfires in the last 20 years^18,19^. This aspect is of particular importance because more pronounced declines in biodiversity are expected in areas exposed to large fires, with long periods without burning (> 5 years), and distant from large, long unburnt vegetation patches^20^. Large unburnt areas are paramount to preserve vertebrate diversity in ecosystems subject to wildfires^21^, especially in less rugged lowlands^20^. Nonetheless, the impacts of wildfires on Brazilian biodiversity are still poorly known^22^, especially on the fauna, preventing our better understanding of large-scale cumulative effects on ecosystem functioning and provision of ecosystem services^13^.

Concerning wildfire impacts on fauna, mammals are a good study case to predict impacts on biodiversity, such as changes in assemblage richness and composition, and species-specific responses to certain aspects of wildfires. Mammals are speciose in the Neotropical realm, particularly in Brazil, to date, comprising 778 species^23^ with a wide range of ecological characteristics that are fundamental to better understanding fire effects on wildlife, such as dietary and habitat specializations, locomotor habits, and species from low to high-mobility. Moreover, some are commonly used as ‘umbrella species’ to protect other taxonomic groups^24^ because of their appealing charismatic nature or important ecological roles^25^. The Pantanal is considered a stronghold for wildlife populations in South American lowlands, containing an assortment of species from distinct biomes, Atlantic Forest, Cerrado, Amazonia, Chiquitana Forest, and Chaco^12^, drawing worldwide attention in response to the catastrophic wildfires of 2020.

To improve our understanding of wildfire impacts on biodiversity, we evaluated the ecological aspects of terrestrial mammals in a protected area and its surroundings in the Brazilian Pantanal, subject to the catastrophic megafire of 2020. Considering the challenges of recording mammals due to their low detectability and elusive habits, we used a combination of methods (camera traps and environmental DNA) to expand their detection success in this study. First, we evaluated modifications in assemblage diversity and composition before and after fire passage, and then, the differences in diversity, composition, and relative abundance between forest types (monospecific vs. polyspecific), burned and unburned sites, and how environmental variables shaped these aspects at the species, site, and assemblage level.

We hypothesize that: i) megafires of high severity, such as in the Pantanal in 2020, tend to result in more animal mortality^4^, reducing species diversity compared to a pre-fire condition; ii) since wildfires can alter habitat structure, which in turn may reduce resource diversity and availability, and shelter opportunities^5^, there will be a decline in species richness and relative abundance, and changes in the composition of mammal assemblages between burned and unburned sites, differences that will increase as burned area proportion increases; iii) polyspecific forests tend to be more resistant to wildfire events^26,27^, therefore, a severe impact is expected for more homogeneous habitats (i.e., monospecific forests), consequently, reducing mammalian diversity and their relative abundance therein.

## Results

### Mammal diversity

**Figure 1:**
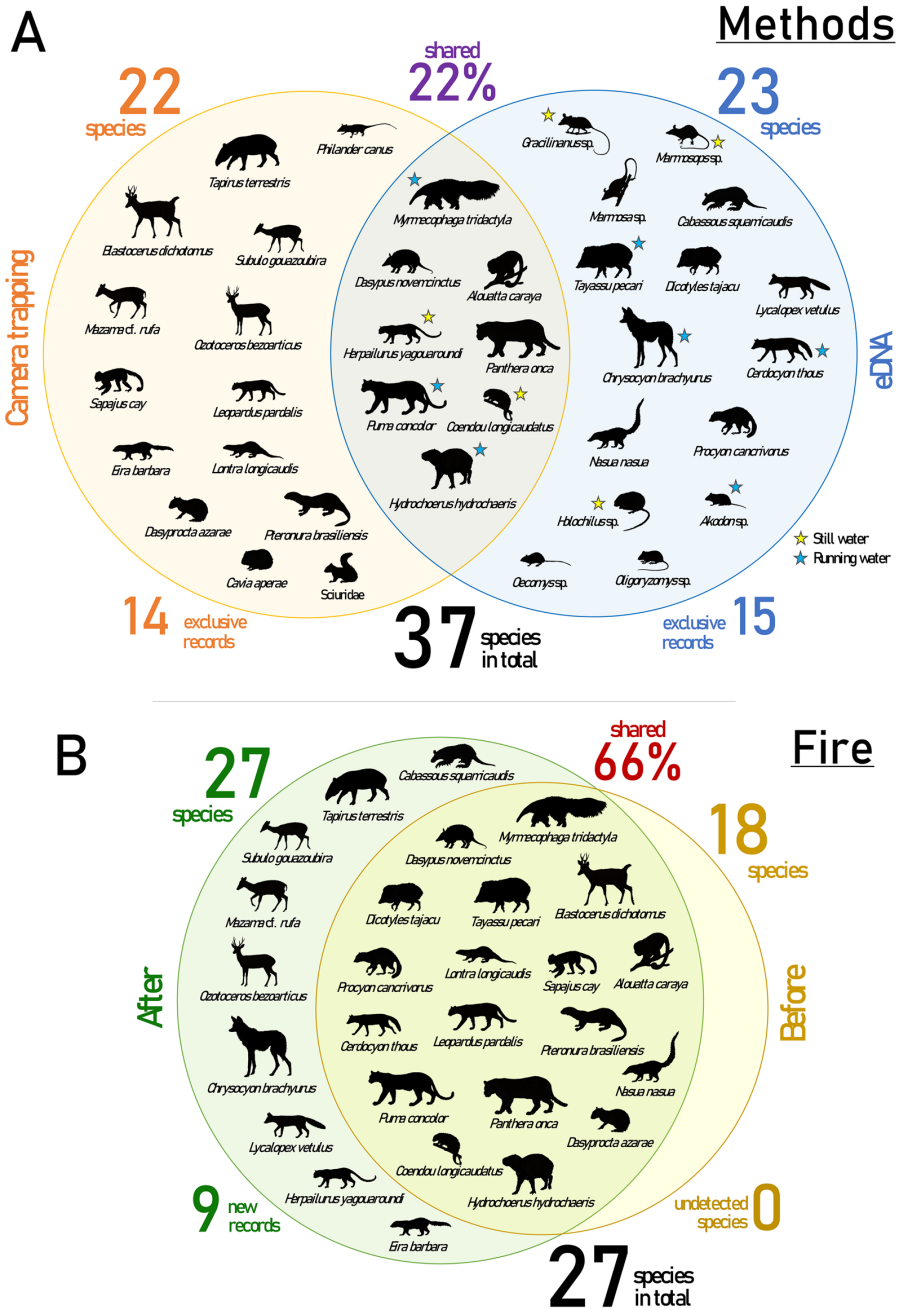
Venn diagrams comparing mammal assemblage composition in Taiamã Ecological Station (TES) and its surrounding areas in northern Pantanal, Cáceres, Mato Grosso, Brazil. (**A**) Comparison between camera trapping and environmental DNA (eDNA) surveys. (**B**) Comparison of medium and large-sized mammal assemblages before^28,29^ and after fire passage (this study).

Combining camera trapping and environmental DNA (eDNA) surveys, we recorded a total of 37 mammal species, including 10 small and 27 medium and large-sized ones, comprising eight orders and 17 families (Supplementary Table 1, Supplementary Fig. 1). Twenty-two species were recorded by camera trapping, with 14 being exclusively recorded by this method, while eDNA recorded 23 mammals (16 identified at species level), with 15 exclusive records (Fig. 1A); methods shared 22% of the species records (N = 8). Eleven species are listed as threatened in Brazil, while six are threatened worldwide (Supplementary Table 1). A detailed summary of eDNA results is available in Supplementary Data 1 and Supplementary Table 2.

### Wildfire effects on assemblage composition

Comparing assemblage composition, considering only medium and large-sized mammals, 18 species were recorded before the fire^28,29^, while 27 were recorded after (this study; camera trapping and eDNA), sharing 66% of all species recorded between periods (N = 18) (Fig. 1B). All species recorded before the fire were detected in our study, while nine were new records: *Cabassous squamicaudis*, *Tapirus terrestris*, *Mazama* cf. *rufa*, *Subulo gouazoubira*, *Ozotoceros bezoarticus*, *Chrysocyon brachyurus*, *Lycalopex vetulus*, *Herpailurus yagouaroundi*, and *Eira barbara*. For a comparison of the assemblage composition among studies, see Supplementary Fig. 2.

### Species, site, and assemblage-level changes

**Figure 2:**
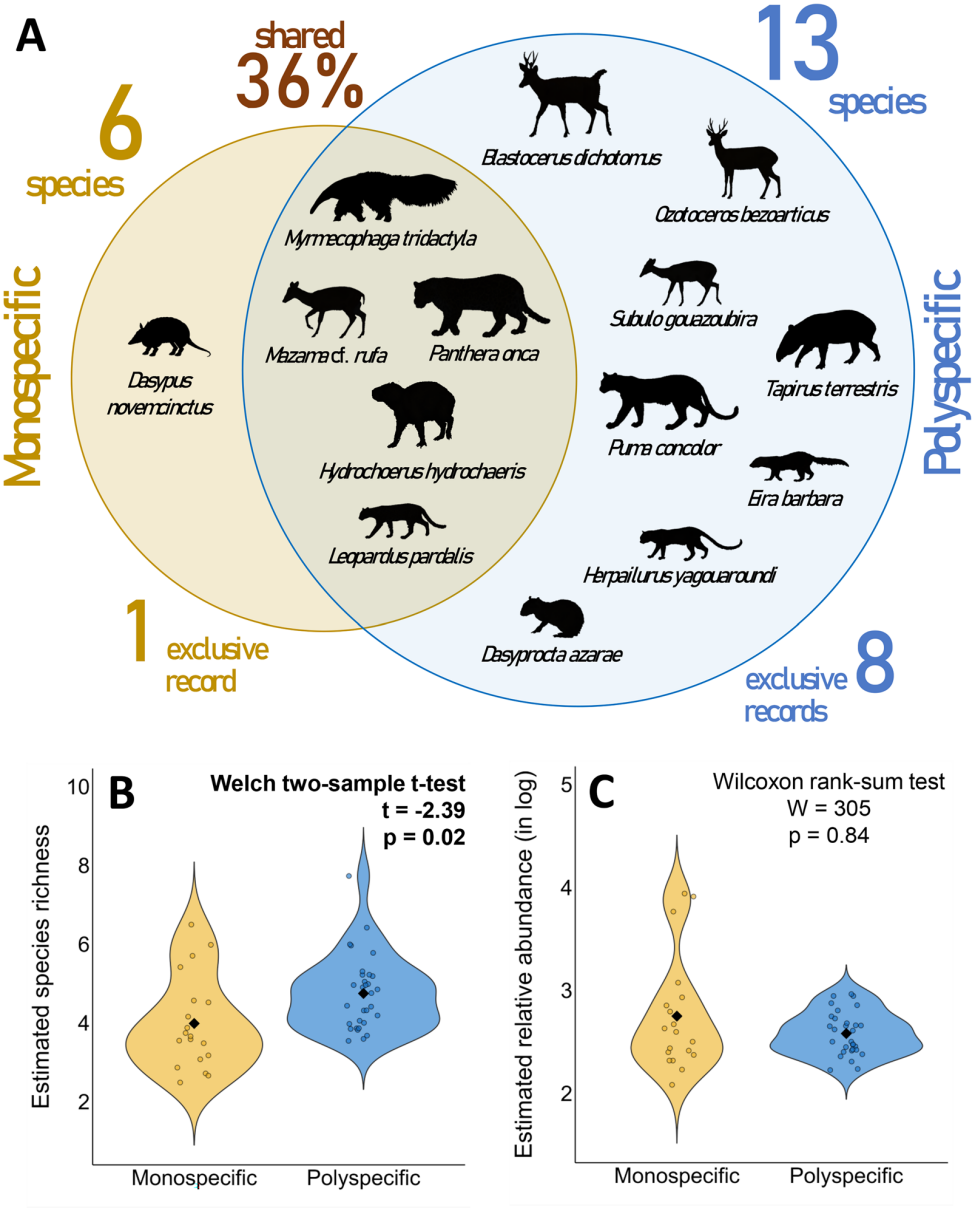
(**A**) Composition of the mammal assemblage between forest types in Taiamã Ecological Station (TES) and its surrounding areas in northern Pantanal, Cáceres, Mato Grosso, Brazil. Variation in the site level mean species richness (**B**) and aggregated relative abundance (**C**) of mammals between forest types estimated by the Bayesian multi-species occupancy model. Values in bold indicate statistical support.

Mammal assemblages shared 36% of species between forest types (Fig. 2A), with higher observed and estimated richness in polyspecific forests (Welch two-sample t-test, t = − 2.39, *p* = 0.02; Fig. 2B), and similar aggregated relative abundance (Wilcoxon rank-sum test, W = 305, *p* = 0.84; Fig. 2C).

**Figure 3:**
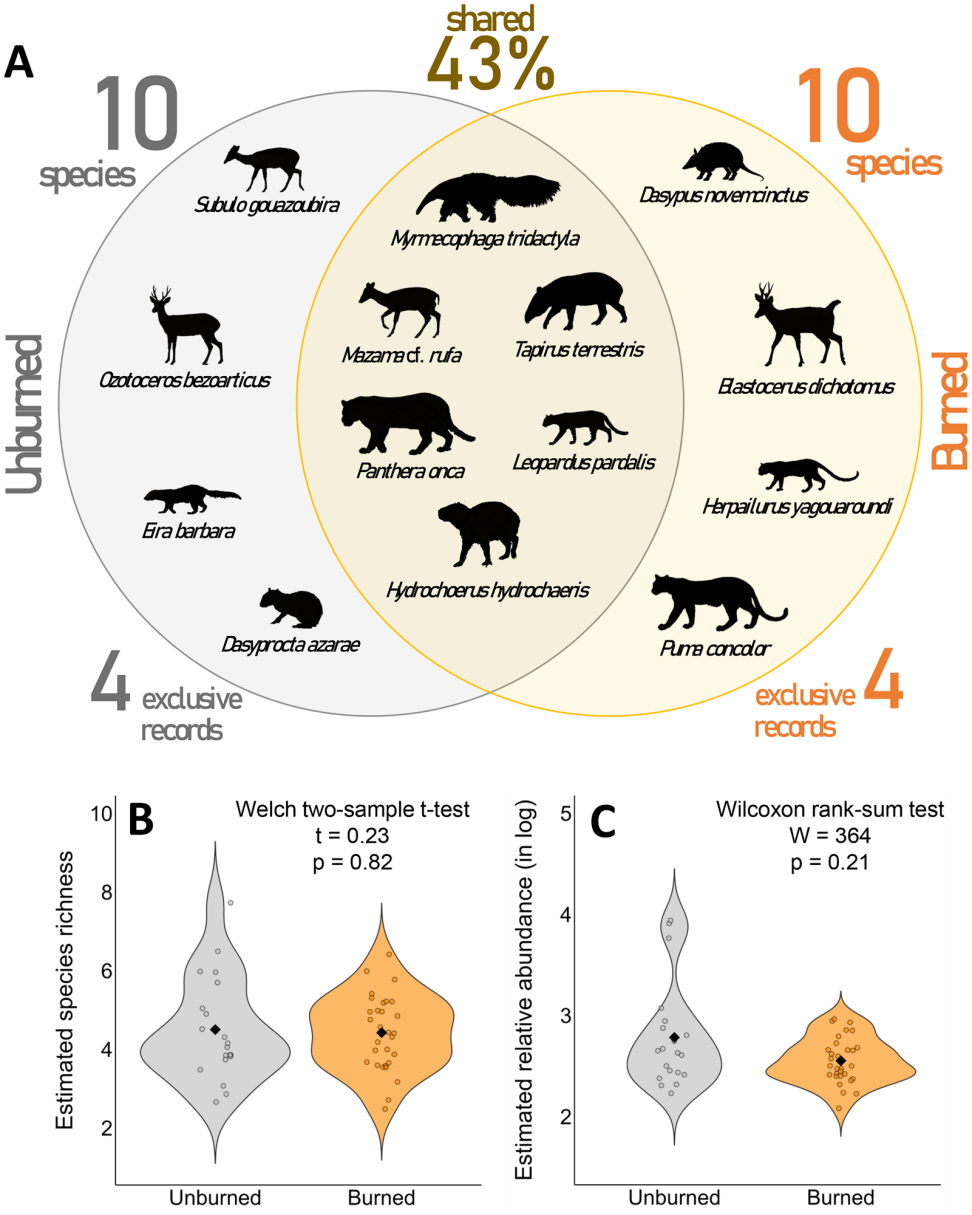
(**A**) Composition of the mammal assemblage between burned and unburned sites in Taiamã Ecological Station (TES) and its surrounding areas in northern Pantanal, Cáceres, Mato Grosso, Brazil. Variation at the site level mean species richness (**B**) and aggregated relative abundance (**C**) of mammals between burned and unburned sites estimated by the Bayesian multi-species occupancy model.

Although the observed species richness was similar between burned and unburned sites, mammal assemblage composition differed, with 43% of species shared and four exclusive records in each treatment (Fig. 3A). Based on the MSOM results, the previous pattern was sustained, with no difference in species richness (Welch two-sample t-test, t = 0.23, *p* = 0.82; Fig. 3B) or aggregated relative abundance at the site level (Wilcoxon rank-sum test, W = 364, *p* = 0.21; Fig. 3C).

**Figure 4:**
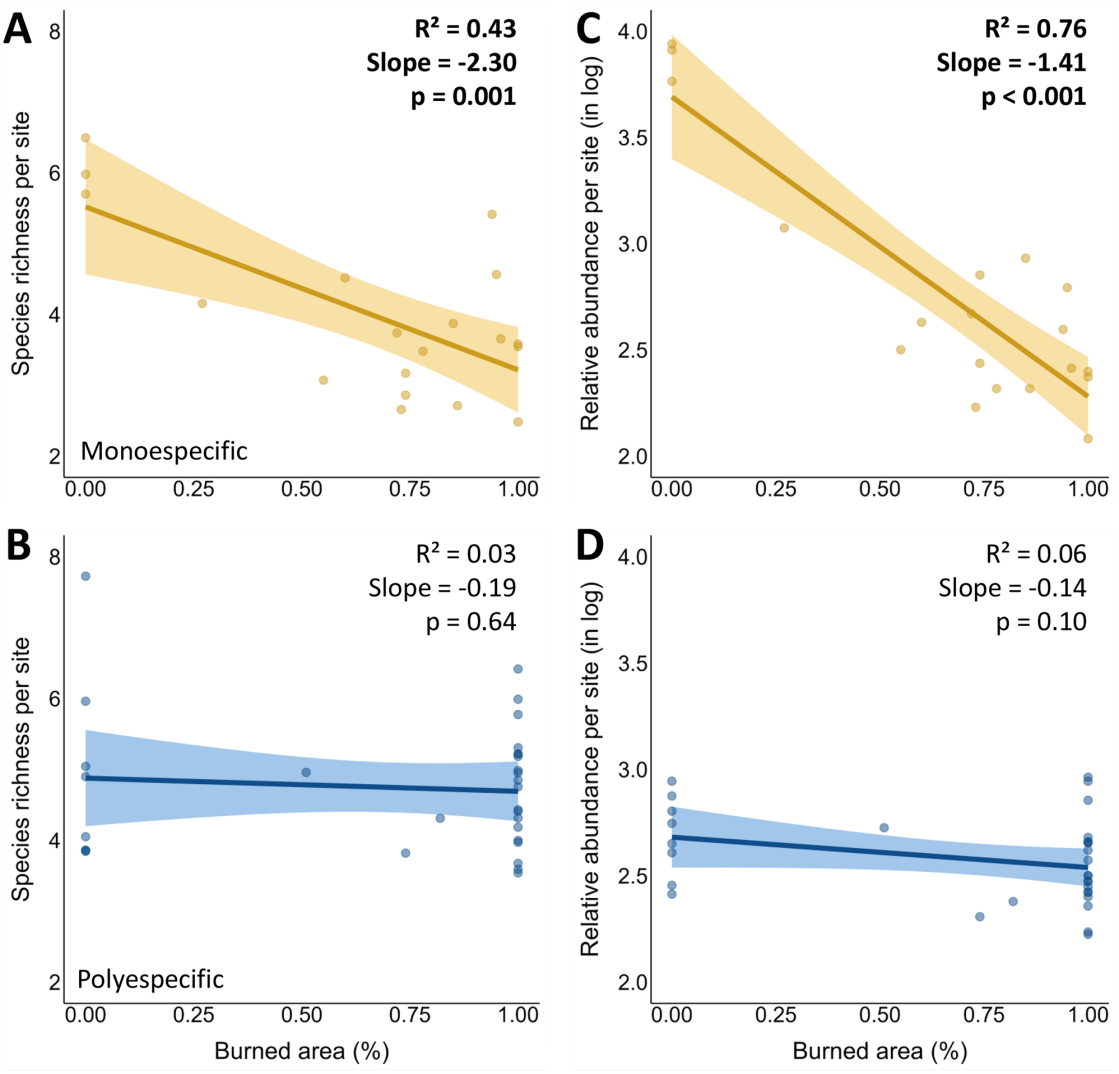
Linear relationships of species richness and aggregated relative abundance per sampling station according to forest type [monospecific (**A**, **C**); polyspecific (**B**, **D**)] estimated by the Bayesian multi-species occupancy model, with the proportion of burned area in Taiamã Ecological Station (TES) and its surrounding areas in northern Pantanal, Cáceres, Mato Grosso, Brazil. Results in bold indicate statistical support.

As the proportion of burned area increased in monospecific forests, mammalian richness (R^2^ = 0.76, slope = − 1.41, *p* < 0.001; Fig. 4A) and aggregated relative abundance (R^2^ = 0.43, slope = − 2.30, *p* = 0.001; Fig. 4C) estimated by the MSOM, presented significant decreases at the site level. Although presenting slight decreases, no substantial changes were observed in the parameters in polyspecific forests (species richness: R^2^ = 0.06, slope = − 0.14, *p* = 0.10; aggregated relative abundance: R^2^ = 0.03, slope = − 0.19, *p* = 0.64; Fig. 4B,D).

**Figure 5:**
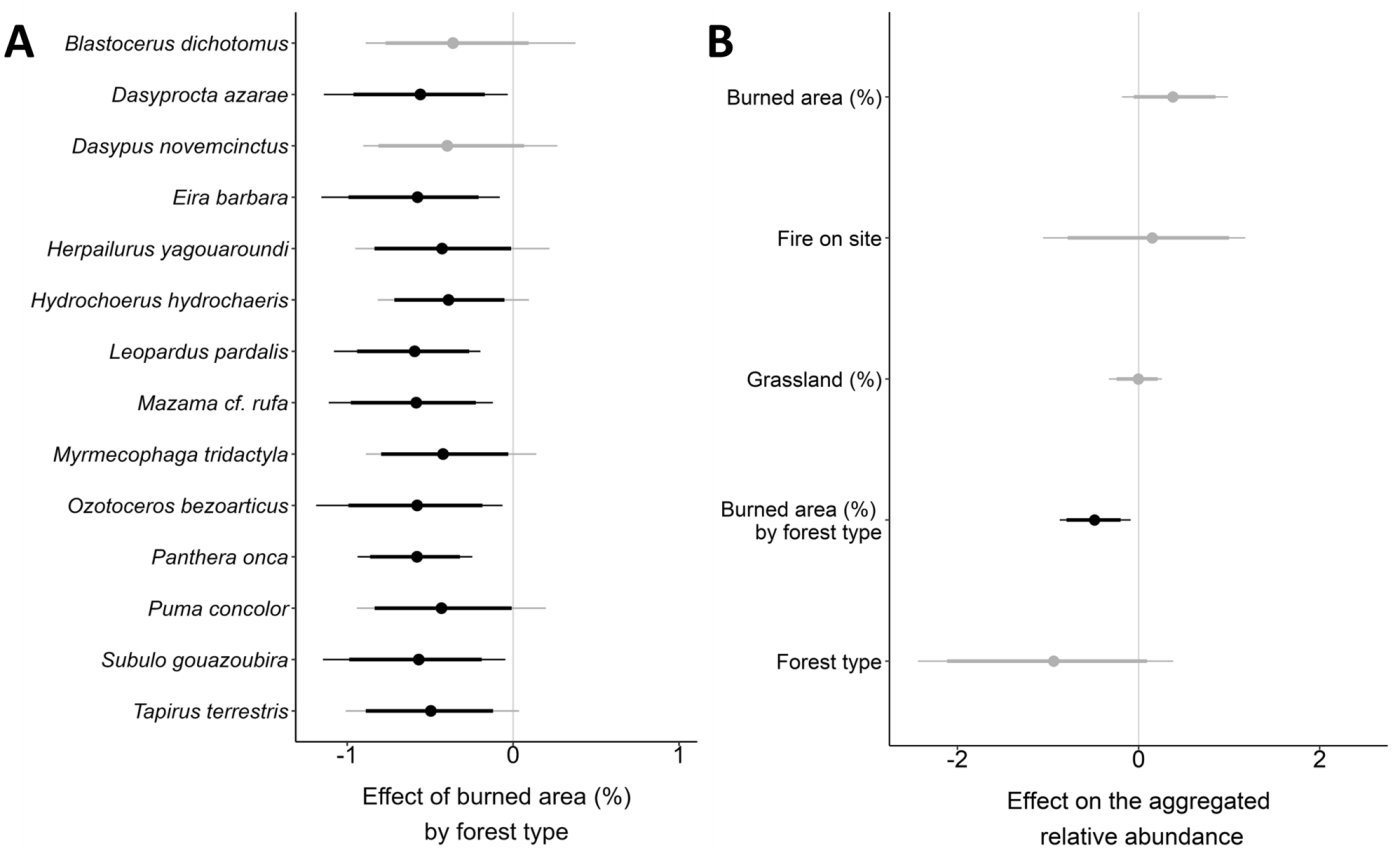
Results of the Bayesian multi-species occupancy model, showing the magnitude and direction (Bayesian means ± credible intervals) for the posterior distributions of the interaction between the proportion of burned area and forest type on the relative abundance of mammals at the species (**A**) and assemblage levels (**B**) in Taiamã Ecological Station (TES) and its surrounding areas in northern Pantanal, Cáceres, Mato Grosso, Brazil. For effects including forest type, negative values = monospecific, positive = polyspecific. For effects including fire on site, negative = burned, positive = unburned. Black lines indicate statistical support (thin = 95%; thick = 90%).

Considering the effects of variables in the MSOM over individual species, only the interactivity between the proportion of burned area and forest type showed significant moderate to strong effects on the relative abundance parameter (Fig. 5A, Supplementary Figs. 3–5). We observed that mammals presented an average reduction in relative abundance in response to increasing burned area proportion in monospecific forests, with a moderate effect for five species and a strong one for seven. A strong effect was also observed for the interactive variable on the assemblage aggregated relative abundance, which decreased in monospecific forests (Fig. 5B).

## Discussion

Assessing biodiversity responses to wildfires is paramount to understanding the impacts of such events on species occurrence and abundance, especially considering that they are predicted to occur more frequently with climate change^30^. Although our study area was subject to a catastrophic megafire in 2020, a year later, the terrestrial mammalian diversity was higher than before the fire. Species previously undetected might have been attracted by habitat modification caused by fires, particularly herbivores and open-area tolerant mammals. Assemblage composition differed between burned and unburned sites, but not species richness or relative abundance. However, partitioning the effect of the proportion of burned area per forest type, we documented a differential response of mammals, from the species to the assemblage level, with an overall decrease in species richness and relative abundance as burned area proportion increased in monospecific forests. Therefore, our hypothesis that monospecific forests are more vulnerable to wildfires than polyspecific ones was confirmed, with a negative consequence for mammalian richness and relative abundance.

The complementarity of camera trapping and eDNA sampling, evidenced by the reduced number of species shared between methods (22%), highlights their reliability to be combined when aiming at richness estimation^31^. Moreover, these methods used together provided a more accurate assessment of the mammalian diversity after fire passage, allowing for the detection of species that otherwise would be missed if only one method was employed. Nineteen mammal species were added to the list of TES and its surrounding areas, increasing the overall richness to 37 species. Therefore, although 38.1% of TES and 70% of its surroundings were severely burned (in a 10-km buffer^16^), mammalian diversity increased compared to a pre-fire condition^28,29^.

These results contradict our first hypothesis, which predicted lower species richness after fire passage. Megafires can substantially alter assemblage composition via habitat modification^5^, for example, favoring the growth of herbaceous and grassy plants, which might attract herbivores^32^, such as those we detected only after fire passage (i.e., *O. bezoarticus*, *M.* cf. *rufa*, *S. gouazoubira*, and *T. terrestris*). For instance, in African savannas and grasslands, and other fire-prone ecosystems like Cerrado, the abundance of large herbivores was positively impacted by fires, although responses vary widely among taxa depending on species life-history traits, habitat requirements, and refuges provided by unburned habitats (e.g.,^7,8,33^).

However, other species occurring in areas surrounding TES, namely *Aotus azarae*, *Cuniculus paca*, *Tamandua tetradactyla*, *Euphractus sexcinctus*, and *Tolypeutes matacus* (recorded by interviews^28^), were not detected in our study. Species with low mobility and habitat specializations are more vulnerable to megafires^8^, such as arboreal/scansorial (i.e., *A. azarae*, *T. tetradactyla*) and semi-fossorial (i.e., *E. sexcinctus* and *T. matacus*) animals, which might explain their non-detection. Similarly, group-living animals, such as peccaries (*Tayassu pecari* and *Dicotyles tajacu*), also tend to present higher mortality in response to megafires^8^, as observed for white-lipped peccaries during data collection in other parts of Pantanal (^13^, C. Berlinck, pers. observ.).

Some species had their DNA detected only in running water samples, namely *Akodon* sp.*, C. thous*, *C. brachyurus*, *M. tridactyla*, *H. hydrochaeris*, *P. concolor*, and *T. pecari*, with the first five classified as open-area tolerant. DNA concentrations in running water can be subject to downstream transport up to 45 km from the sampling point, considering a river flow equal to 300 m^3^/s and DNA persistence of ~ 12 h at 30 °C^34^. Therefore, samples collected in running water may contain DNA from species occurring upstream of the study area, in our case, *C. brachyurus,* not expected to occur in this region^35^. Nonetheless, *L. vetulus*, a species also not expect for the region^36^, was recorded in still water. In this sense, as mentioned above, some species might be attracted to altered environments created by wildfires, particularly open-area tolerant and diet generalist ones, which might explain the presence of the aforementioned species.

It is noteworthy to mention that wildfire impacts on small mammals are yet virtually unknown. Tomas et al.^13^ estimated that small rodents and marsupials summed up about 20% of the dead animals in Pantanal, which might be underestimated. Nevertheless, studies in the Cerrado showed that some generalist small mammals may experience an increase in abundance after wildfires or are poorly affected, while habitat specialists may take months or years to recover or reappear (e.g.,^37–39^). In this study, we detected both habitat generalist (*Akodon* sp., *Oligoryzomys* sp.) and specialist (*Gracilinanus* sp., *Marmosa* sp., *Marmosops* sp., *Holochilus* sp., *Oecomys* sp.) small mammal about 1 year after the fires. Nonetheless, the lack of previously published studies on small mammals in the study area prevents further comparisons.

It is difficult to uncover the aspects regarding the presence of small mammals after the megafire, particularly considering their detection by eDNA from water samples, which hinders record association to forest type or burned/unburned sites. Nevertheless, some species present mechanisms of response to wildfires^40^, allowing individuals to survive, which may play an important role in the recovery of small mammal populations locally^41^. For example, Semedo et al.^42^ reported individuals of marsh rat (*Holochilus chacarius*) found alive inside partially flooded underground burrows just after the megafires in Pantanal. Therefore, further studies focused on home range estimation, use of space, and genetics may contribute to a better understanding of the effects of wildfires on small mammals.

Species richness was higher in polyspecific forests for both observed and estimated richness, highlighting their resistance, to some extent, to wildfires^26,27^, even during catastrophic events, something observed in other heterogeneous fire-prone ecosystems (e.g.,^43^). Despite differences in assemblage richness and composition, the aggregated relative abundance was similar between forest types, indicating a distinct landscape use by the species. Only assemblage composition differed between burned and unburned sites, confirming one aspect of our second hypothesis, which predicted changes in species richness and relative abundance, and assemblage composition after fire passage. This response was observed for forest-dependent and open-area tolerant species, strengthening that mammals may respond differently to fire conditions based on their life history traits (e.g.,^20,33^).

The strong effects of burned area proportion on monospecific forests led to pronounced reductions in mammalian richness and aggregate relative abundance at the site level and promoted significant decreases in relative abundance at the species and assemblage levels as the proportion of burned areas increased. Eighty-six percent of the species presented moderate to strong negative effects on their relative abundance, including forest-dependent (e.g., *T. terrestris*, *P. concolor*) and open-area tolerant mammals (e.g., *S. gouazoubira*, *O. bezoarticus*), although five out of seven forest-dependent species were strongly affected (e.g., *D. azarae*, *E. barbara*, *L. pardalis*, *M.* cf. *rufa*, *P. onca*). The negative effect at the assemblage level stresses the vulnerability of monospecific forests to megafires, which might result in long-term impacts on mammalian abundance besides direct mortality, that is, related to a prolonged reduction in food resources diversity and availability, and shelter opportunities, following our third hypothesis that predicted more severe impacts at this forest type.

This result might be a consequence of the habitat simplification caused by megafires of great severity^44^, which tend to reduce mammalian abundance varying with the recovery time of the vegetation^7^. Although *Erythrina fusca* monospecific forests seem to be favored by local environmental factors, such as low soil fertility and higher flood levels^45^, the complete mischaracterization of the environment by the megafire (Supplementary Fig. 6), slowed and even prevented their recovery. The aerial structures that allow the colonization of flooded areas by monospecific forests, also make them more susceptible to wildfire (C. Berlinck, pers. observ.). In this forest type, the 2020 megafire penetrated more than 1 m below ground, sterilizing the dry histosol, burning up the organic matter, the soil seed bank, and the underground structures of plants such as roots^46^, resulting in drastic environmental changes and, consequently, long-term negative effects for mammals and other taxa.

Seven species had their relative abundance strongly affected by increasing burned area proportion in monospecific forests, of which, two are threatened at the national level^47^, the Pampas deer (*O. bezoarticus*) and the jaguar (*P. onca*). Populations of these large species are generally small and isolated throughout their distribution, with the Pantanal being a stronghold for both species in Brazil. The Pantanal 2020 megafire highlighted the negative impacts on less mobile and habitat specialist taxa, which are expected to be more affected, and high-mobile species like the jaguar^13^. A current assessment of this megafire impacts on jaguar populations indicates a disturbing scenario for the long-term conservation of the species considering the proportion of individuals and habitats potentially affected^48^.

Drastic declines in biodiversity are more associated with large fires, fire-prone vegetation not burned for long periods, and large distances between burnt and large unburnt vegetation patches^20^; the two former aspects are observed in TES and its surrounding areas. More than one-third of the study area was affected by the 2020 megafire^16^, leaving some unburnt forest patches that possibly acted as refugia for the high-mobile mammal species in the reserve and its surroundings. Large unburnt areas and forest canopy preservation are crucial to support vertebrate survival after megafires^20,21,43^, especially for diet and habitat specialist taxa, such as those recorded in this study.

Here, we presented evidence that forest type modulates fire severity, inducing changes in mammalian richness, composition, and relative abundance in response to a catastrophic megafire. We acknowledge the limitation of not having a measure of the species’ relative abundance for the period before the fire for a more meticulous comparison. However, we stress the perceived impact of this single incident at several levels of organization, from species to the assemblage, highlighting its severe and widespread negative consequences. Wildfire activity and its impacts on biodiversity are being transformed by anthropogenic drivers, such as climate and land use changes^30^, tending to increase conditions that favor wildfires in the Pantanal in the forthcoming years^49^. Cumulative effects, frequency, and severity of wildfires are responsible for reducing the diversity and abundance of organisms^4,5,7,43^^.^ If such events, analogous to the Pantanal 2020 megafire, become recurrent, they might trigger local and regional extinctions in a short time.

Management strategies such as prescribed fire become crucial to prevent and reduce the negative impacts of future wildfire events. This technique, among other management strategies, proves beneficial to fire-prone ecosystems, fostering biodiversity, nutrient cycling, and reducing the risk of catastrophic events^50^. Advancements in fire science, allied with remote sensing and climate data available in large temporal and spatial scales, and high computational power coupled with artificial intelligence, produced systems able to detect and prevent fire events, such as in the Brazilian Cerrado^51^. For the Pantanal, specifically, there is the ALARMES system (https://alarmes.lasa.ufrj.br/), a platform that monitors fires and their spreading by combining satellite imagery, hotspots, and artificial intelligence to support environmental agencies in actions to combat wildfires. Importantly, these practices also help mitigate negative repercussions on wildlife species by creating a mosaic of habitats, offering refuges, and preventing extensive habitat alteration. These approaches acknowledge the intricate balance between natural fire regimes and human efforts to safeguard ecosystems and their biodiversity.

## Methods

### Study area

**Figure 6:**
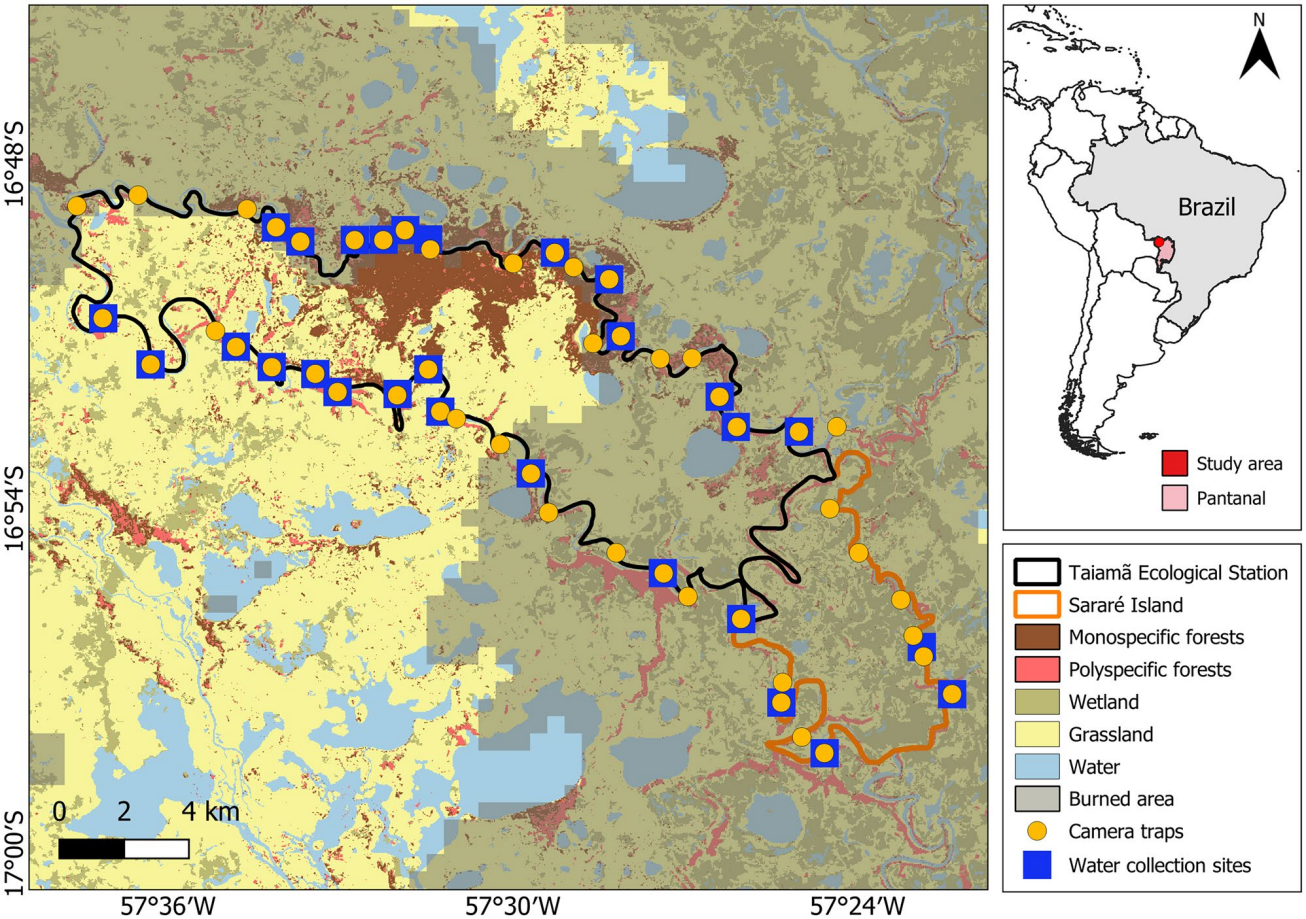
Location of Taiamã Ecological Station (TES) and surrounding areas in northern Pantanal, Cáceres, Mato Grosso, Brazil, showing the location of the camera trapping stations (N = 50), water sample sites (N = 28), forest types^52^, areas burnt in 2020^54^, and other landuses^55^. Generated with QGIS 3.34.2 (https://qgis.org/).

The study was conducted in a protected area – Taiamã Ecological Station (TES) – and its surrounding areas in northern Pantanal, Cáceres, Mato Grosso, Brazil (Fig. 6). TES is an island delimited by the Paraguay River and its branch (locally named Bracinho River), comprising an area of 115.55 km^2^. Southeast of TES lies Sararé Island (31.25 km^2^), which is part of a proposal for creating a new protected area. TES and its surrounding areas are mainly composed of floodplains, containing a great variety of aquatic environments, such as permanent, temporary, and meander lagoons, and ‘corixos’ (i.e., natural connections between rivers and lagoons) (Supplementary Fig. 7). According to the Köppen classification, the climate is characterized as AW, consisting of two seasons (wet and dry) with average annual temperature ranging between 20 and 32 °C, and average precipitation of 1500 mm year-round.

The study area macro-habitats include aquatic macrophyte fields (48% of the island), flooded fields (24%), monospecific (16%) and polyspecific forests (8%), and lakes (4%)^52^. Polyspecific forests are formed by shrubs and pioneer forests along riverbanks (Supplementary Fig. 8). The monospecific forest, locally known as ‘abobral’, is composed of individuals of *Erythrina fusca* Lour (Fabaceae), which is a dominant pioneer tree and occurs sparsely in riparian forests, mainly in Pantanal and Amazon (Supplementary Fig. 6). This forest type is associated with floating histosol^45^, which is rich in organic matter and can colonize areas that are seasonally flooded longer than polyspecific ones^53^.

### Droughts and wildfire events

According to Marengo et al.^11^, the drought events observed in Pantanal between 1962–1965 and 1967–1972 registered high severity, similar to 2018–2020. Regarding the 1962–1972 period, drought intensity was higher in November 1962, while for 2018–2020, drought was higher in April 2020, the driest month since 1900. The main cause of the lack of rainfall during the consecutive summers of 2019 and 2020, was a change in the South American monsoon system, which reduced the seasonal transport of water vapor from the Amazon basin into the Pantanal during the austral summer. This reduced accumulated rainfall caused severe drought conditions, creating favorable conditions for wildfire spread in the central-western/northern Pantanal^56^. A large forest fire impacted TES and its surrounding areas in 2011^55^, and although other wildfire events occurred in the region from the year 2000 onwards, they did not affect the study areas (http://terrabrasilis.dpi.inpe.br/queimadas/bdqueimadas/).

### Field data acquisition

We classified mammals in three body size categories [small (< 1 kg), medium (from 1 to 7 kg), and large (> 7 kg)^57,58^], as forest-dependent or open-area tolerant, and according to their diet and locomotor type^25,59^. We followed the list of the Brazilian Society of Mammalogy^23^ as the taxonomic authority, and assigned threat categories at national^47^ and international levels^60^. We used specialized literature to identify mammal species^57,61–63^. Data collection was authorized by SISBIO #79,107–1 and SisGen #ABF0E81 permits.

#### Camera trapping

From August to November 2021, a total of 55 sampling stations were deployed in TES and its surrounding areas, with a mean distance of 1 km from each other, following the TEAM protocol^64^ (Fig. 6). A single unbaited camera trap (Bushnell, models 119949C and 119932C; Browning models Patriot and SPEC OPS ELITE HP4) was installed per station at ~ 40 cm above the ground, programmed to take three photos at 0.6 s intervals between bursts, and operating 24 h/day. We considered sampling stations independent if they were at least 500 m from each other; those within this range had their records combined, which resulted in 50 independent stations. Cameras were active from 92 to 99 days, totaling a sampling effort of 4639 trap-days. Sampling stations were distributed following two treatments: forest type, divided into monospecific (N = 19) and polyspecific (N = 31), and in burned (N = 20) and unburned sites (N = 30) (Supplementary Figs. 2 and 3). Forest type was determined according to Frota et al.^52^. Burned sites were determined by the area burned in a 30 m buffer from the sampling station location during fieldwork. We used the web platform Wildlife Insights (https://www.wildlifeinsights.org/) to store, organize, and identify all focal species records.

#### Environmental DNA (eDNA)

We used water as an environmental sample to survey mammals by metabarcoding sequencing. Water collection was conducted in lentic and lotic water bodies near the camera trapping stations, with other points sampled when those were not available. We classified water samples as still water when collected in ponds or puddles (N = 7), and running water when collected in rivers or streams (N = 21), totaling 28 collection points (Fig. 6). The eDNA was obtained by water sample filtration. The collected water was poured into a syringe, and the plunger was placed and pushed manually at a flow rate of 1 mL for 10 s for filtration^65^. A volume of 45 mL was passed through a polyethersulfone membrane filter (0.22-µm pore size, 30 mm diameter, Kasvi) using a sterilized disposable syringe of 20 mL. All sampling equipment was handled with clean latex gloves, which were changed among sampling sites, and the equipment was cleaned with a 10% bleach solution after each collection. The filters were transported refrigerated at ~ 0 °C and the membranes were removed from the filters and stored in one milliliter of Longmire buffer^66^ at − 20 °C.

The membranes were vortexed for 30 s and 400 μL of the buffer was removed for extraction. DNA extraction was performed in a room dedicated to processing low-quantity DNA samples using the DNeasy PowerWater Kit (Qiagen). We amplified two mini-barcode regions from ribosomal mitochondrial genes (12S and 16S rRNA) using primers previously described for targeting vertebrates (12SV5F and 12SV5R^67^) and mammals (16Smam1 and 16Smam2^68^), respectively. We used a dual index strategy, where the product of PCR1 was cleaned using magnetic beads (Agencourt AMPure XP® – Beckman Coulter), quantified using a Qubit fluorometer (Thermo Fisher, Waltham, Massachusetts, USA), normalized to a concentration of 20 ng/µL and indexed using a Nextera Index kit® (Illumina, San Diego, California, USA). The paired-end sequencing was performed on the Illumina iSeq® platform, using an iSeq v2 300 Cycle Reagent kit (2 × 150 bp).

The bioinformatics pipeline was organized in R 4.3.1^69^. In brief, reads were initially submitted to remove undetermined bases, and quality filtering (Q-scores ≥ 30). Only reads containing the expected index sequence corresponding to each sample were kept for subsequent analysis. Error correction, read-pair merging, and chimera identification and removal were performed using the default settings of DADA2 functions^70^. No length truncation was performed since the primer removal step automatically clips uninformative regions and resulting ASVs (Amplicon Sequence Variants) out of the expected amplicon length range for each marker (135–139 bp for 12SrRNA, and 130–134 bp for 16SrRNA) were discarded. Subsequently, identified ASVs were clustered into OTUs (Operational Taxonomic Units) using SWARM v3.1.0^71^, applying the fastidious option and d = 1. Taxonomic assignments were conducted using alignment of the NCBI nucleotide collection using an automated BLAST + 2.10.1 function with minimum similarity and minimum coverage (-perc_identity 90 and -qcov_hsp_perc 90). The OTUs were also compared with sequences available in GenBank for species identification using the BLAST tool (https://blast.ncbi.nlm.nih.gov/Blast.cgi).

The final dataset included only OTUs with > 90% similarity against the GenBank database and containing > 5 reads (0.5% relative abundance). All taxonomic assignments were manually curated. When a sequence had a match for two or more species with equal similarity, we selected those with expected occurrence in the studied area. When a high percentage of matches was obtained (≥ 98%), but the species is not expected to occur in the Pantanal biome, we assigned the genus. This situation generally happens when the mini-barcode sequence from the species is unavailable in GenBank and matched with other species of the same genus (e.g., small mammals). We also assumed the genus assignment for sequence matches between 90 and 97.99%.

### Landscape variables

We calculated the main land uses in TES and its surrounding areas using the land use and land cover map of MapBiomas, collection 7, for year 2021^55^, and the package *landscapemetrics*^72^. Land uses were classified into three categories: forest formations (codes 3 and 4), grassland (code 12), and water (codes 11 and 33), which were calculated in 1-km circular buffers from the center of each sampling station. After calculation, we performed a Pearson correlation, in which forest and water presented a positive correlation (> 0.5) and, thus, were removed from subsequent analyses. Using the map of Pinto et al.^54^, we calculated the proportion of burned area in 1 km circular buffers from the center of each sampling station.

### Data analysis

All analyses were performed in R 4.3.1, and graphical implementation was done using the *ggplot2* package^73^.

#### Mammal diversity and wildfire effects on assemblage composition

Considering all species recorded in TES and its surrounding areas, we compared the richness and composition of mammal assemblages between survey methods (camera trapping and eDNA) using a Venn diagram. Then, focusing solely on medium and large-sized mammals (> 1 kg), we compared the richness and composition of the assemblages in a pre-fire condition (hereafter “before the fire”) using data from de Lázari et al.^28^ and Eriksson et al.^29^, and after fire passage (this study), employing a Venn diagram.

#### Species, site, and assemblage-level changes

To evaluate species-specific responses, and site and assemblage-level changes in mammalian richness and relative abundance, by using camera trap data, we performed a Bayesian multi-species occupancy model (MSOM), assuming that our sampling design and temporal replicates followed the major model assumptions described by Devarajan et al.^74^. We created detection histories per sampling station based on detection and non-detection records over 5-day intervals for all species using the *detectionHistory* function of the *camtrapR* package^75^, and then, fitted a Royle-Nichols MSOM following Yamaura et al.^76^. The MSOM framework assumes that individuals of species *i* at site *j* are independently detected with probability *r*_*ij*_ and the detection probability for a species at a site depends on the local abundance of that species according to:

1 $$p_{ij} = { 1 } - \, \left( {{1 } - r_{ij} } \right)^{Zij}$$

where *p*_*ij*_ is the detection probability of species *i* at site *j*, while *Z*_*ij*_ is the abundance of species *i* at site *j. Y*_*ij*_ is the detection frequency of species over *V*_*ij*_ visits and follows a binomial distribution with parameter *p*_*ij*_ (*Y*_*ij*_ ~ Binomial (*Vj*, *p*_*ij*_)), and the local population size *Z*_*ij*_ follows a Poisson distribution whereby mean *λ*_*ij*_ (*Z*_*ij*_ ~ Poisson (*λ*_*ij*_)), and both *r*_*ij*_ and *λ*_*ij*_ can be modeled by the explanatory variables.

Our model included five explanatory variables affecting the abundance parameter at each sampling station, comprising burned and unburned sites (Local_fire), the forest type (polyspecific or monospecific; Forest_type), the proportion of grassland (Grassland), the proportion of burned area in 1-km buffers (Burned_area), and the interaction between forest type and burned area proportion, as follows:

2 $$\begin{aligned} {\text{log}}\left( {\lambda_{ij} } \right) \, = & {\text{ a}}0_{i} + {\text{ a1}}_{i} {\text{Local}}\_{\text{fire}}_{j} + {\text{ a2}}_{i} {\text{Forest}}\_{\text{type}}_{j} + {\text{ a3}}_{i} {\text{Grassland}}_{j} + {\text{ a4}}_{i} {\text{Burned}}\_{\text{area}}_{j} \\ & + {\text{a5}}_{i} {\text{Forest}}\;{\text{type}}_{j} *{\text{ Burned}}\;{\text{area}}_{j} \\ \end{aligned}$$

We modeled the individual detection probability parameter to depend on burned and unburned sites, forest type, and the number of camera traps per sampling station (N_camera_traps), as follows:

3 $${\text{logit}}\left( {r_{ij} } \right) \, = \, \beta 0_{i} + \, \beta {1}_{i} {\text{Local}}\_{\text{fire}}_{j} + \, \beta {2}_{i} {\text{Forest}}\_{\text{type}}_{j} + \, \beta {3}_{i} {\text{N}}\_{\text{camera}}\_{\text{traps}}_{j}$$

In our MSOM, the local abundance parameters *Z*_*ij*_ represent the number of individuals available for detection around each sampling station^77^, and they can usefully represent a relative measure of the intensity of species habitat use^78^, especially for the larger species for which one individual can be detected by more than one sampling station^79^. We used a data augmentation procedure, adding all-zero detection histories for eight “potentially” undetected species, to estimate species richness at the site level (see^80^). These eight ‘dummy’ species were determined based on species occurrence available in studies before^28,29^ and after fire passage (this study). Refer to Supplementary Table 1 for the complete species list. We computed (i) the posterior mean species richness and (ii) the aggregated posterior means of the relative abundance of each species per site (hereafter*,* relative abundance).

From all of the species recorded with camera trapping, we removed seven whose records are not favored by ground camera trapping (i.e., arboreal and semi-aquatic species, and small mammals), except for *Hydrochoerus hydrochaeris*, which was detected throughout TES and its surrounding areas. Therefore, we included 14 recorded mammals plus eight ‘dummy’ species, totaling 22 species in the analysis. The model was fitted by three Markov Chain Monte Carlo using the *rjags* package^81^. We used non-informative priors for all parameters, with 100,000 iterations per chain, a burn-in of 50,000, and a thinning rate of 100. We evaluated chain convergence using the Gelman-Rubin convergence diagnostic (Rhat < 1.05^82^), assuming an adequate accuracy in parameter estimation when the effective sample size was larger than 100 per parameter^83^, besides making visual inspections of trace plots to confirm convergence. We considered evidence of support for an explanatory variable when the estimated posterior credible interval did not include zero, as follows: strong (≥ 95%), moderate (≥ 90%), and weak (< 90%) effects. The model script is available in Supplementary Data 2.

After calculations, we compared the observed species richness and assemblage composition between forest types and burned and unburned sites, considering the 14 species used in the MSOM. Then, using the posterior estimates generated by the MSOM, we compared the average species richness and the aggregate relative abundance per sampling station between forest types and burned and unburned sites. Welch's two-sample t-test and Wilcoxon rank-sum test were employed depending on data normality, which was assessed using the Shapiro–Wilk test. Next, using linear regressions, we assessed the relationship between species richness and aggregate relative abundance estimated by the MSOM for each sampling station, with the proportion of burned area per forest type. Finally, we assessed the magnitude and direction effects of the explanatory variables used in the MSOM on the relative abundance and detection of each species, as well as for the entire assemblage.

### Supplementary Information

Supplementary Information.

Supplementary Table 2.

## Data Availability

Data used in the analyses is available in Mendeley Data (https://data.mendeley.com/datasets/9kf63xkt9y/1).
