# Supplementary material for: Forest type modulates mammalian responses to megafires
Source: Sci Rep. 2024 Jun 12;14:13538. doi: 10.1038/s41598-024-64460-3 (PMC11169498; doi:10.1038/s41598-024-64460-3)
Supplement: Supplementary file 1 — Supplementary Information. [file 41598_2024_64460_MOESM1_ESM.docx]

Supplementary material for:

**Forest type modulates mammalian responses to megafires**

Marcelo Magioli^1,2,3^, Luanne Helena Augusto Lima^2^, Priscilla Marqui Schmidt Villela^4^, Ricardo Sampaio^2,3^, Lilian Bonjorne^2^, Renan Lieto Alves Ribeiro^2^, Daniel Luis Zanella Kantek^5,6^, Selma Samiko Miyazaki^5,6^, Thiago B. F. Semedo^7,8,9^, Gustavo S. Libardi^10^, Bruno H. Saranholi^11^, Charlotte E. Eriksson^12^, Ronaldo Gonçalves Morato^2,13^, Christian Niel Berlinck^2^

^1^ Instituto Pró-Carnívoros, Atibaia, São Paulo, Brazil; ^2^ Centro Nacional de Pesquisa e Conservação de Mamíferos Canívoros, Instituto Chico Mendes de Conservação da Biodiversidade, Atibaia, São Paulo, Brazil; ^3^ Laboratório de Ecologia e Conservação (LAEC), Departamento de Biologia, Faculdade de Filosofia, Ciências e Letras de Ribeirão Preto (FFCLRP), Universidade de São Paulo, Ribeirão Preto, Brazil; ^4^ EcoMol Consultoria, Piracicaba, São Paulo, Brazil; ^5^ Estação Ecológica de Taiamã, Chico Mendes Institute for the Conservation of Biodiversity, Cáceres, Mato Grosso, Brazil; ^6^ Centro Nacional de Pesquisa e Conservação de Mamíferos Aquáticos, Instituto Chico Mendes de Conservação da Biodiversidade, Santos, São Paulo, Brazil; ^7^ CIBIO, Centro de Investigação em Biodiversidade e Recursos Genéticos, InBIO Laboratório Associado, Campus de Vairão, Universidade do Porto, 4485-661 Vairão, Portugal; ^8^ BIOPOLIS Program in Genomics, Biodiversity and Land Planning, CIBIO, Campus de Vairão, 4485-661 Vairão, Portugal; ^9^ Departamento de Biologia, Faculdade de Ciências, Universidade do Porto, 4099-002 Porto, Portugal; ^10^ Facultad de Ciencias Exactas, Físicas y Naturales, Universidad Nacional de Córdoba, Córdoba, Argentina; ^11^ Departamento de Genética e Evolução, Universidade Federal de São Carlos, São Carlos, Brazil; ^12^ Department of Fisheries and Wildlife, Oregon State University, Corvallis, Oregon 97331 USA; ^13^ Departamento de Conservação e Uso Sustentável da Biodiversidade, Secretaria Nacional de Biodiversidade, Floresta e Direito dos Animais, Ministério do Meio Ambiente e Mudança Clima, Brasília, Brazil

**Supplementary data 1**

*eDNA summary*

We obtained a total of 4,066,595 paired reads after sequencing and quality filtering, of which 312,596 were of mammal species (discarding human), with a mean number of reads equal to 795.4 ± 999.2 per OTUs for 12S rRNA (range: 5-11,527) and 257.2 ± 228.2 for 16S rRNA (range: 6-7,073). For 12S rRNA, we also retrieved 3,753,999 paired reads from other non-mammal vertebrates. A total of 190,150 reads from domestic or exotic species (i.e., *Bos taurus, Canis lupus familiaris, Sus scrofa, Felis catus*, and *Rattus norvergicus*) were also detected, and 100,048 reads from mammal species that could not be identified (match <90% of similarity). Concerning small mammals (< 1 kg), some sequences matched with species that do not occur or are not reported in Pantanal (*Gracilinanus microtarsus*, *Marmosa* (*Micoureus*) *demerarae*, and *Marmosops* *pinheiroi*), while others matched with congeneric species in the biome and surroundings (*Akodon montensis*, *Oecomys mamorae*, and *Oligoryzomys chacoensis*). Nonetheless, the taxonomic representativeness of 12S and 16S rRNA available in public databases was insufficient to cover the whole biodiversity of the area and provide species-level assignments, as already pointed out in other studies with mammals^1,2^. As eDNA combined with metabarcoding sequencing is a recent methodology used in biodiversity inventory and monitoring, efforts to produce such reference sequences are still needed^1,3^.

**Supplementary data 2**

*Script of the Bayesian multi-species occupancy model*

## ----- Specify model in JAGS language -----

sink(file = "RN.model.formula.aug-taiama-inter.txt")

cat("model {

# Prior distributions on community level estimates - hyperparameters

psi ~ dunif(0, 1) # Inclusion rate that generates w[i]

# Mean value (mu)

# Parameters related to relative abundance

mu.a0 ~ dnorm(0, 0.01) # intercept on lambda

mu.a1 ~ dnorm(0, 0.01) # slope on lambda for Forest_type

mu.a2 ~ dnorm(0, 0.01) # slope on lambda for Local_fire

mu.a3 ~ dnorm(0, 0.01) # slope on lambda for Grassland

mu.a4 ~ dnorm(0, 0.01) # slope on lambda for Burned_area

mu.a5 ~ dnorm(0, 0.01) # slope on lambda for Forest_type * Burned_area

# Parameters related to detectability

mu.r0 ~ dnorm(0, 0.01) # intercept on lambda

mu.r1 ~ dnorm(0, 0.01) # slope on lambda for Forest_type

mu.r2 ~ dnorm(0, 0.01) # slope on lambda for Local_fire

mu.r3 ~ dnorm(0, 0.01) # slope on N_camera_traps

# Standard deviation

# Parameters related to relative abundance

sigma.a0 ~ dunif(0, 10) # intercept

sigma.a1 ~ dunif(0, 10) # Forest_type

sigma.a2 ~ dunif(0, 10) # Local_fire

sigma.a3 ~ dunif(0, 10) # Grassland

sigma.a4 ~ dunif(0, 10) # Burned_area

sigma.a5 ~ dunif(0, 10) # interaction Forest_type * Burned_area

# Parameters related to detectability

sigma.r0 ~ dunif(0, 10) # intercept

sigma.r1 ~ dunif(0, 10) # Forest_type

sigma.r2 ~ dunif(0, 10) # Local_fire

sigma.r3 ~ dunif(0, 10) # N_camera_traps

# Create precision

# Parameters related to relative abundance

tau.a0 <- pow(sigma.a0, -2) # intercept

tau.a1 <- pow(sigma.a1, -2) # Forest_type

tau.a2 <- pow(sigma.a2, -2) # Local_fire

tau.a3 <- pow(sigma.a3, -2) # Grassland

tau.a4 <- pow(sigma.a4, -2) # Burned_area

tau.a5 <- pow(sigma.a5, -2) # interaction Forest_type * Burned_area

# Parameters related to detectability

tau.r0 <- pow(sigma.r0, -2) # intercept

tau.r1 <- pow(sigma.r1, -2) # Forest_type

tau.r2 <- pow(sigma.r2, -2) # Local_fire

tau.r3 <- pow(sigma.r3, -2) # N_camera_traps

for(i in 1:(nspecies + nzeros)) {

# Generating parameters for each species related to relative abundance

a0[i] ~ dnorm(mu.a0, tau.a0) # intercept

a1[i] ~ dnorm(mu.a1, tau.a1) # Forest_type

a2[i] ~ dnorm(mu.a2, tau.a2) # Local_fire

a3[i] ~ dnorm(mu.a3, tau.a3) # Grassland

a4[i] ~ dnorm(mu.a4, tau.a4) # Burned_area

a5[i] ~ dnorm(mu.a5, tau.a5) # interaction Forest_type * Burned_area

# Generating parameters for each species related to individual detection

r0[i] ~ dnorm(mu.r0, tau.r0) # intercept

r1[i] ~ dnorm(mu.r1, tau.r1) # Forest_type

r2[i] ~ dnorm(mu.r2, tau.r2) # Local_fire

r3[i] ~ dnorm(mu.r3, tau.r3) # N_camera_traps

# Indicator variable whether each species is exposed to sampling or not

w[i] ~ dbern(psi)

# Likelihood - Ecological model for latent abundance of species i in sites j

for(j in 1:nSites){

# Population abundances

log(lambda[j,i]) <- a0[i] + a1[i]* Forest_type[j] + a2[i]* Local_fire[j] + a3[i]* Grassland[j] +

a4[i]* Burned_area[j] + a5[i]* Forest_type[j]* Burned_area[j]

Z[j,i] ~ dpois(lambda[j,i]) # Latent abundance of each species in each site

A[j,i] <- Z[j,i] * w[i] # Latent abundance only for extant species

o[j,i] <- step(A[j,i]-1) # Occupancy of each species in each site

# Detection process model

r[j,i] <- 1/(1+exp(-(r0[i] + r1[i]* Forest_type[j] + r2[i]* Local_fire[j] + r3[i]* N_camera_traps[j])))

p[j,i] <- 1-pow(1-r[j,i], A[j,i])

y[j,i] ~ dbin(p[j,i], k[j]) # Model observation data as binomial outcome

} #j

} #i

## Counting species richness at site

for(j in 1:nSites){

SR[j] <- sum(o[j,])

## Counting abundance at site

AB[j] <- sum(A[j,])

}}", fill=TRUE)

sink()

| **Supplementary Table 1.** Mammalian species recorded in Taiamã Ecological Station (TES) and its surrounding areas in northern Pantanal, Cáceres, Mato Grosso, Brazil, depicting their mean body mass (kg), dietary and locomotor habits, forest dependency (FD) or open-area tolerance (OAT)^4,5^, threat categories at national^6^ and international levels^7^, number of records in camera traps (CT), and those detected by environmental DNA (eDNA). Records in grey were obtained by de Lázari et al.^8^. In = insectivore; Om = omnivore; Fr = frugivore; Hb = herbivore; Ca = carnivore; VU = vulnerable; EN = endangered. | | | | | | | | |
| --- | --- | --- | --- | --- | --- | --- | --- | --- |
| **Taxon** | **Body mass** | **Diet** | **Locomotor habit** | **Habitat use** | **Threat category** | | **CT** | **eDNA** |
|  |  |  |  |  | **Brazil** | **World** |  |  |
| DIDELPHIMORPHIA |  |  |  |  |  |  |  |  |
| DIDELPHIDAE |  |  |  |  |  |  |  |  |
| *Gracilinanus* sp. Gardner & Creighton, 1989 | 0.02 | In/Om | Arboreal | FD |  |  |  | x |
| *Marmosa* sp. Gray, 1821 | 0.03 | In/Om | Scansorial | FD |  |  |  | x |
| *Marmosops* sp. Matschie 1916 | 0.04 | In/Om | Scansorial | FD |  |  |  | x |
| *Philander canus* (Osgood, 1913) | 0.05 | In/Om | Scansorial | FD |  |  | 1 |  |
| PILOSA |  |  |  |  |  |  |  |  |
| MYRMECOPHAGIDAE |  |  |  |  |  |  |  |  |
| *Myrmecophaga tridactyla* Linnaeus, 1758 | 30.50 | In | Terrestrial | OAT | VU | VU | 8 | x |
| *Tamandua tetradactyla* (Linnaeus, 1758) | 5.20 | In | Scansorial | FD |  |  |  |  |
| CINGULATA |  |  |  |  |  |  |  |  |
| CHLAMYPHORIDAE |  |  |  |  |  |  |  |  |
| *Cabassous squamicaudis* (Lund, 1845) | 3.20 | In | Semi-fossorial | OAT |  |  |  | x |
| *Euphractus sexcinctus* (Linnaeus, 1758) | 5.40 | In/Om | Semi-fossorial | OAT |  |  |  |  |
| *Tolypeutes matacus* (Desmarest, 1804) | 1.10 | In/Om | Semi-fossorial | OAT |  |  |  |  |
| DASYPODIDAE |  |  |  |  |  |  |  |  |
| *Dasypus novemcinctus* Linnaeus, 1758 | 3.65 | In | Semi-fossorial | FD |  |  | 1 | x |
| PERISSODACTYLA |  |  |  |  |  |  |  |  |
| TAPIRIIDAE |  |  |  |  |  |  |  |  |
| *Tapirus terrestris* (Linnaeus, 1758) | 260.00 | Fr/Hb | Terrestrial | FD | VU | VU | 7 |  |
| ARTIODACTYLA |  |  |  |  |  |  |  |  |
| CERVIDAE |  |  |  |  |  |  |  |  |
| *Blastocerus dichotomus* (Illiger, 1815) | 115.00 | Hb | Terrestrial | OAT | VU | VU | 14 |  |
| *Mazama* cf*. rufa* (Illiger, 1815) | 36.00 | Fr/Hb | Terrestrial | FD |  |  | 4 |  |
| *Subulo gouazoubira* (Fischer, 1814) | 21.00 | Fr/Hb | Terrestrial | OAT |  |  | 1 |  |
| *Ozotoceros bezoarticus* (Linnaeus, 1758) | 35.00 | Hb | Terrestrial | OAT | VU |  | 1 |  |
| TAYASSUIDAE |  |  |  |  |  |  |  |  |
| *Dicotyles tajacu* (Linnaeus, 1758) | 26.00 | Fr/Hb | Terrestrial | FD |  |  |  | x |
| *Tayassu pecari* (Link, 1795) | 35.00 | Fr/Hb | Terrestrial | FD | VU | VU |  | x |
| PRIMATES |  |  |  |  |  |  |  |  |
| AOTIDAE |  |  |  |  |  |  |  |  |
| *Aotus azarae* (Humboldt, 1811) | 1.25 | Hb/Om | Arboreal | FD |  |  |  |  |
| ATELIDAE |  |  |  |  |  |  |  |  |
| *Alouatta caraya* (Humboldt, 1812) | 6.00 | Hb/Fr | Arboreal | FD |  |  | 31 | x |
| CEBIDAE |  |  |  |  |  |  |  |  |
| *Sapajus cay* (Illiger, 1815) | 1.50 | Fr/Om | Arboreal | FD | VU | VU | 6 |  |
| CARNIVORA |  |  |  |  |  |  |  |  |
| CANIDAE |  |  |  |  |  |  |  |  |
| *Cerdocyon thous* (Linnaeus, 1766) | 6.50 | Om | Terrestrial | OAT |  |  |  | x |
| *Chrysocyon brachyurus* (Illiger, 1815) | 25.00 | Om | Terrestrial | OAT | VU |  |  | x |
| *Lycalopex vetulus* (Lund, 1842) | 4.00 | In/Om | Terrestrial | OAT | VU |  |  | x |
| FELIDAE |  |  |  |  |  |  |  |  |
| *Herpailurus yagouaroundi* (É. Geoffroy Saint-Hilaire, 1803) | 4.50 | Ca | Terrestrial | OAT | VU |  | 1 | x |
| *Leopardus pardalis* (Linnaeus, 1758) | 9.50 | Ca | Terrestrial | FD |  |  | 42 |  |
| *Panthera onca* (Linnaeus, 1758) | 109.00 | Ca | Terrestrial | FD | VU |  | 220 | x |
| *Puma concolor* (Linnaeus, 1771) | 45.00 | Ca | Terrestrial | FD |  |  | 1 | x |
| MUSTELIDAE |  |  |  |  |  |  |  |  |
| *Eira barbara* (Linnaeus, 1758) | 7.00 | Ca/Om | Terrestrial | FD |  |  | 1 |  |
| *Lontra longicaudis* (Olfers, 1818) | 6.00 | Ca/Om | Semi-aquatic | FD |  |  | 35 |  |
| *Pteronura brasiliensis* (Zimmermann, 1780) | 29.00 | Ca | Semi-aquatic | FD | VU | EN | 1 |  |
| PROCYONIDAE |  |  |  |  |  |  |  |  |
| *Nasua nasua* (Linnaeus, 1766) | 5.10 | Om | Terrestrial | FD |  |  |  | x |
| *Procyon cancrivorus* (G. Cuvier, 1798) | 5.40 | Om | Scansorial | FD |  |  |  | x |
| RODENTIA |  |  |  |  |  |  |  |  |
| CAVIIDAE |  |  |  |  |  |  |  |  |
| *Cavia aperea* Erxleben, 1777 | 0.55 | Hb | Terrestrial | OAT |  |  | 1 |  |
| *Hydrochoerus hydrochaeris* (Linnaeus, 1766) | 50.00 | Hb | Semi-aquatic | OAT |  |  | 168 | x |
| CUNICULIDAE |  |  |  |  |  |  |  |  |
| *Cuniculus paca* (Linnaeus, 1766) | 9.30 | Fr/Hb | Terrestrial | FD |  |  |  |  |
| CRICETIDAE |  |  |  |  |  |  |  |  |
| *Akodon* sp. Meyen, 1833 | 0.04 | In/Om | Terrestrial | OAT |  |  |  | x |
| *Holochilus* sp. Brandt, 1835 | 0.11 | Fr/Hb | Semi-aquatic | OAT |  |  |  | x |
| *Oecomys* sp. Thomas, 1906 | 0.03 | Fr | Arboreal | FD |  |  |  | x |
| *Oligoryzomys* sp. Bangs, 1900 | 0.03 | Fr | Scansorial | OAT |  |  |  | x |
| DASYPROCTIDAE |  |  |  |  |  |  |  |  |
| *Dasyprocta azarae* Lichtenstein, 1823 | 2.90 | Fr/Hb | Terrestrial | FD |  |  | 4 |  |
| ERETHIZONTIDAE |  |  |  |  |  |  |  |  |
| *Coendou longicaudatus* Daudin, 1802 | 1.80 | Hb | Arboreal | FD |  |  | 2 | x |
| SCIURIDAE |  |  |  |  |  |  |  |  |
| Sciuridae n.i. | 0.17 | Fr/Hb | Arboreal | FD |  |  | 1 |  |


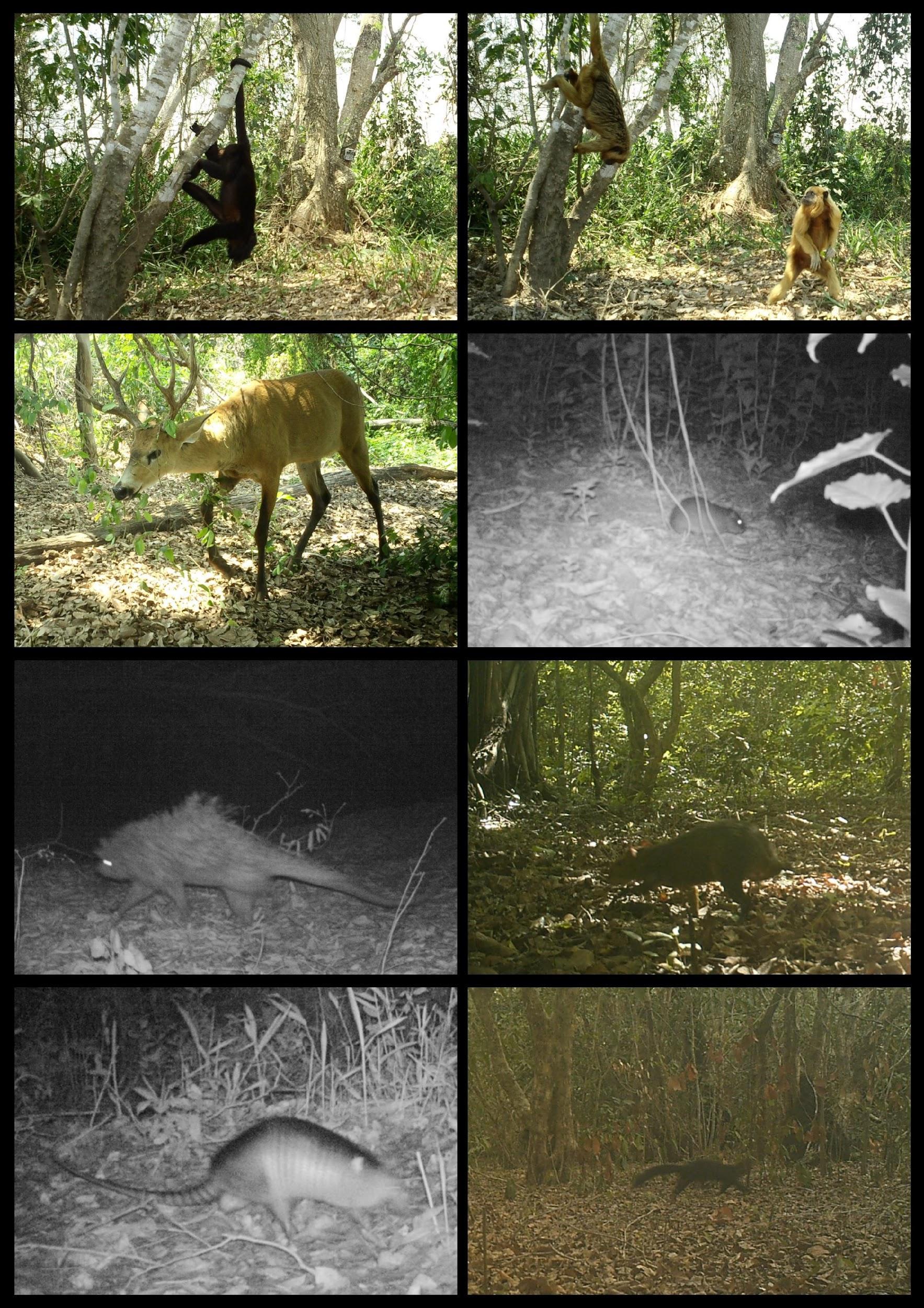


**A**

**B**

**C**

**D**

**E**

**F**

**G**

**H**

**Supplementary Figure 1. (continues)** (A, B) *Alouatta caraya*, (C) *Blastocerus dichotomus*, (D) *Cavia aperea*, (E) *Coendou longicaudatus*, (F) *Dasyprocta azarae*, (G) *Dasypus novemcinctus*, (H) *Eira barbara*.


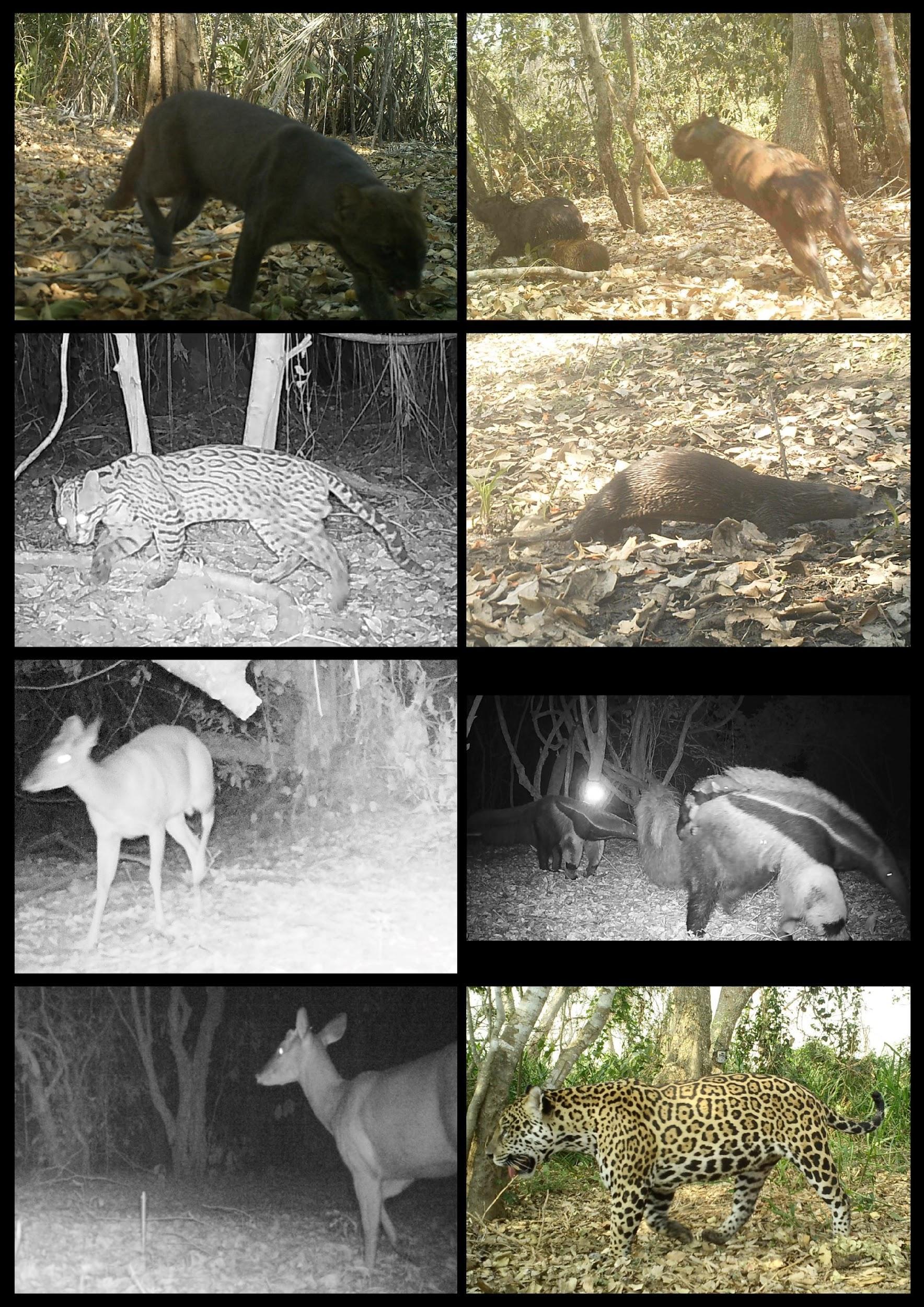


**I**

**J**

**K**

**L**

**M**

**N**

**O**

**P**

**Supplementary Figure 1. (continues)** (I) *Herpailurus yagouaroundi*, (J) *Hydrochoerus hydrochaeris*, (K) *Leopardus pardalis*, (L) *Lontra longicaudis*, (M) *Mazama* cf. *rufa*, (N) *Myrmecophaga tridactyla*, (O) *Ozotoceros bezoarticus*, (P) *Panthera onca*.


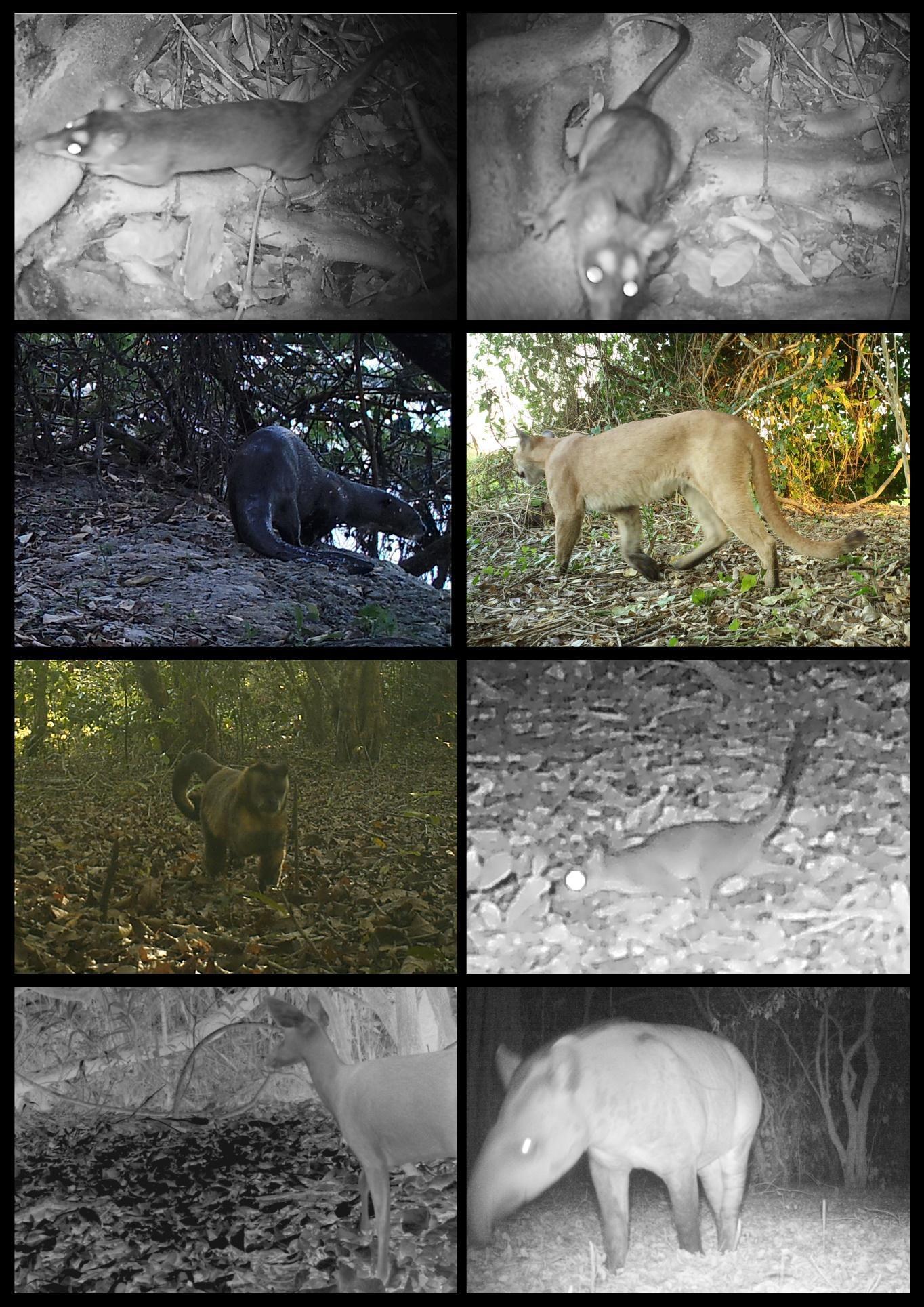


**Q**

**R**

**S**

**TT**

**U**

**V**

**X**

**W**

**Supplementary Figure 1. (end)** (Q, R) *Philander canus*, (S) *Pteronura brasiliensis*, (T) *Puma concolor*, (U) *Sapajus cay*, (V) Sciuridae, (X) *Subulo gouazoubira*, (W) *Tapirus terrestris*.


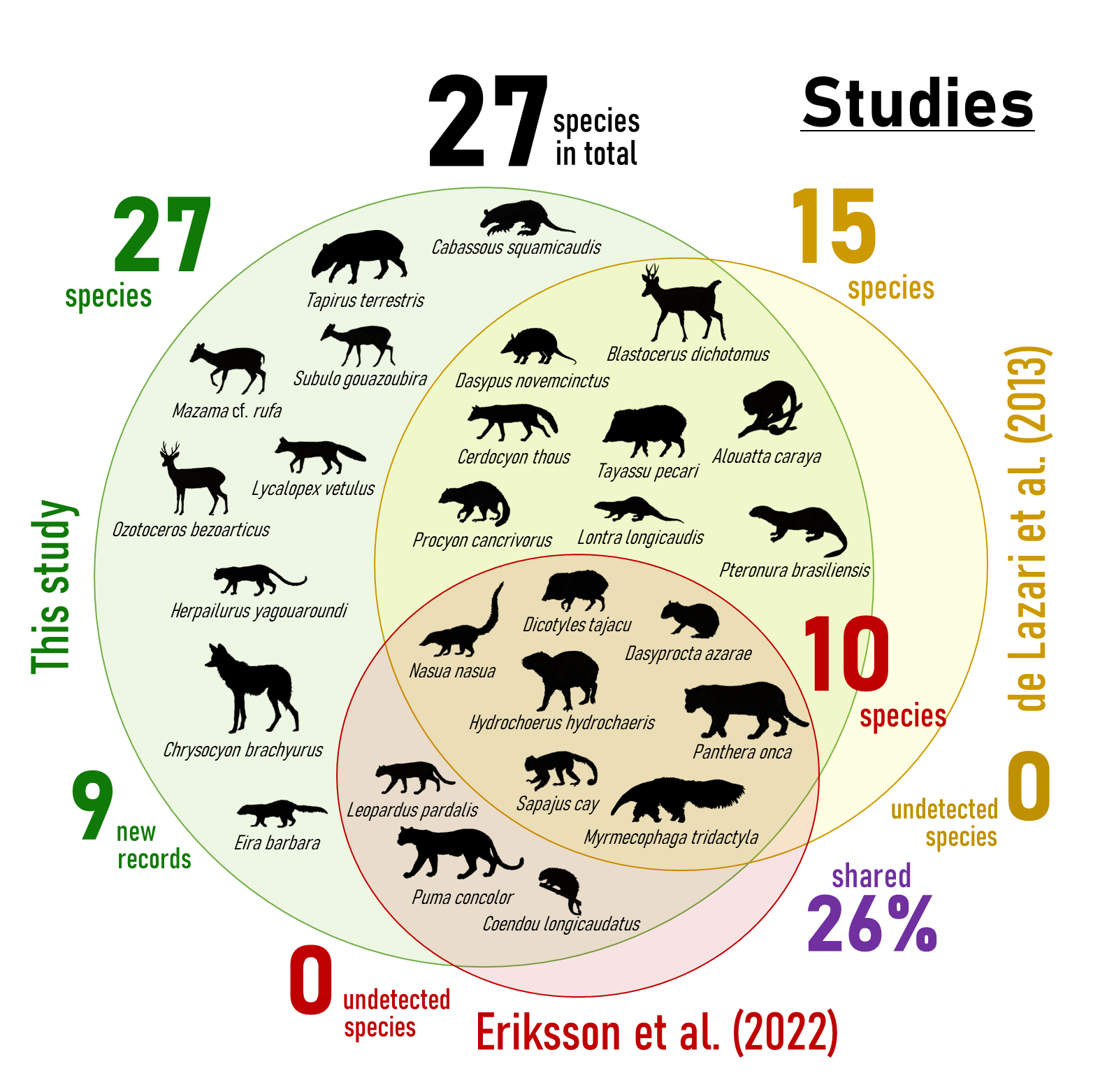


**Supplementary Figure 2.** Venn diagram comparing medium and large-sized mammal assemblages in Taiamã Ecological Station (TES) and its surrounding areas in northern Pantanal, Cáceres, Mato Grosso, Brazil, obtained before the fire by de Lázari et al.^8^ and Eriksson et al.^9^, and after the fire by this study.


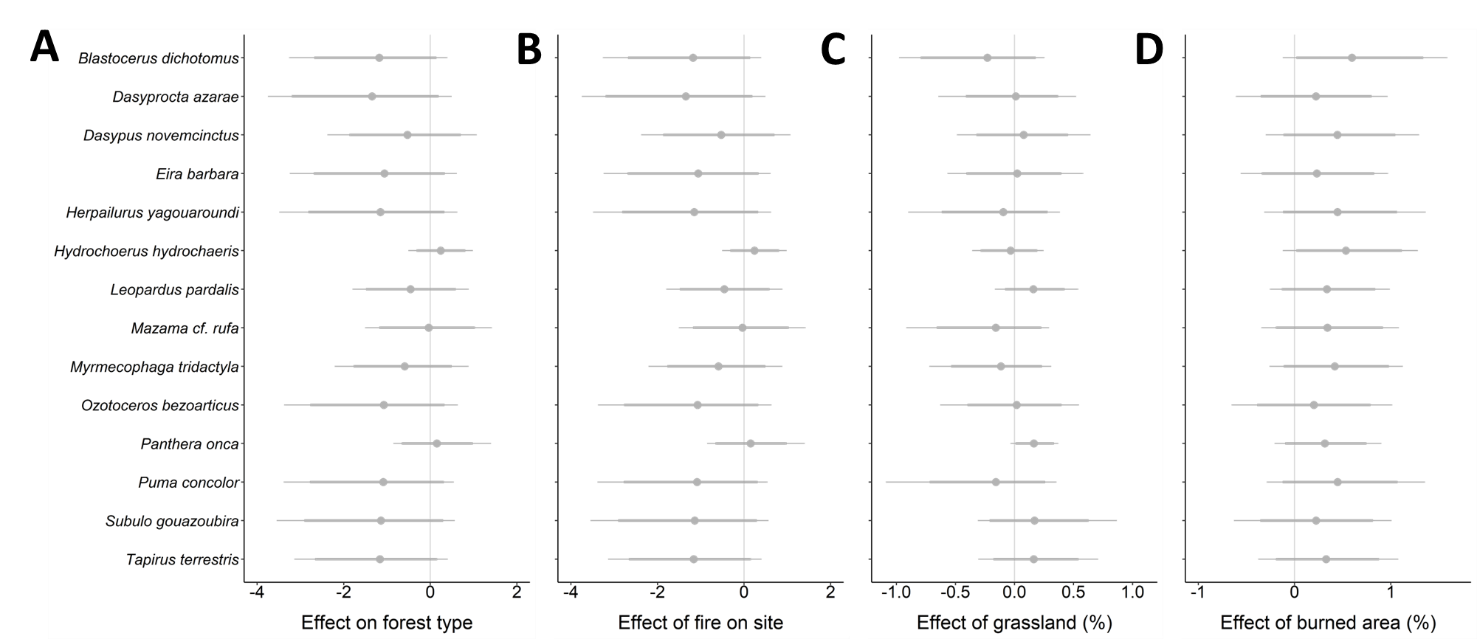


**Supplementary Figure 3.** Results of the Bayesian multi-species occupancy model, showing the magnitude and direction (Bayesian means ± credible intervals) for the posterior distributions of explanatory variables on mammal relative abundance at the species level in Taiamã Ecological Station (TES) and its surrounding areas in northern Pantanal, Cáceres, Mato Grosso, Brazil. For effects including forest type, negative values = monospecific, positive = polyspecific. For effects including fire on site, negative = burned, positive = unburned. Thin line = 95% credible interval, thick line = 90% credible interval.


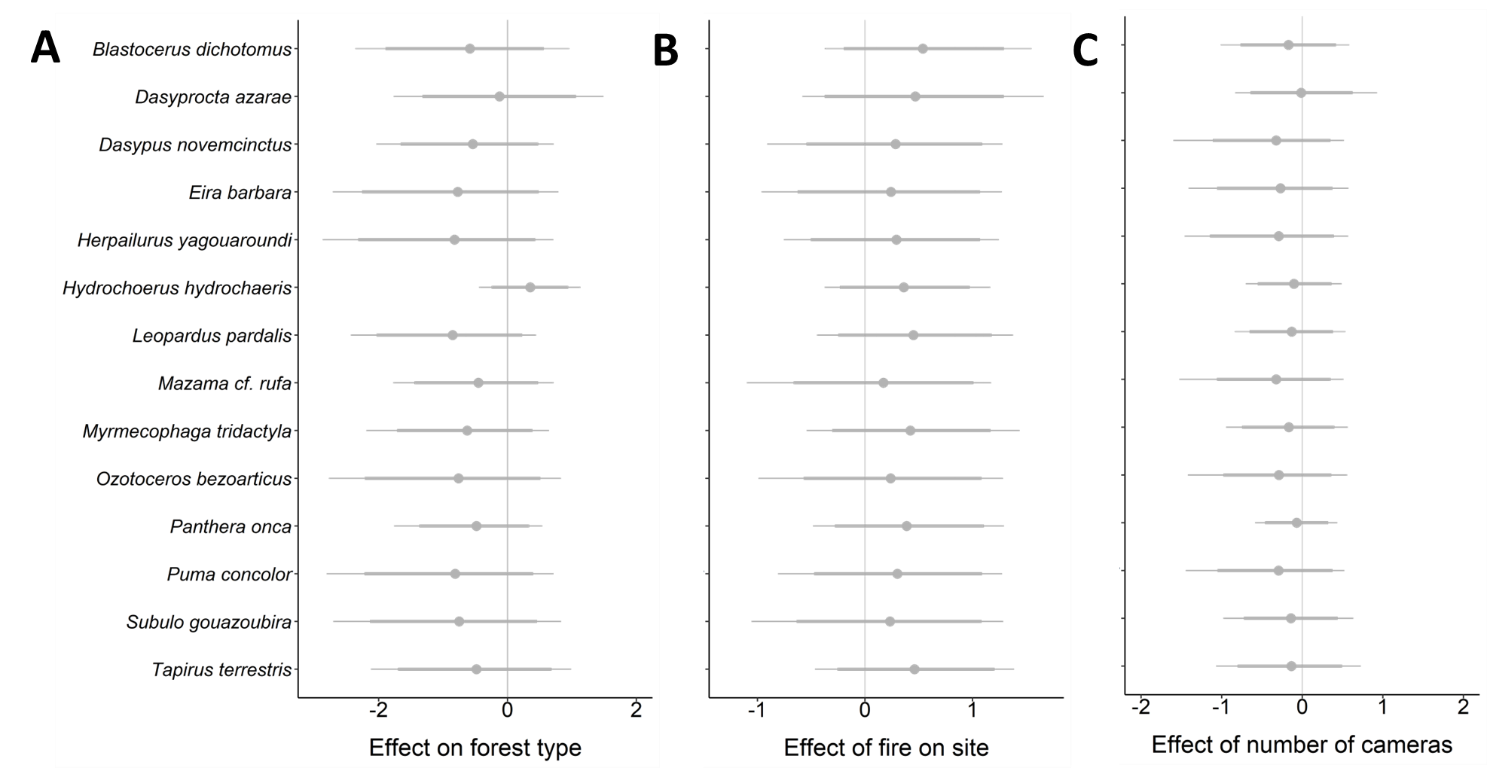


**Supplementary Figure 4.** Results of the Bayesian multi-species occupancy model, showing the magnitude and direction (Bayesian means ± credible intervals) for the posterior distributions of explanatory variables on mammal detection at the species level in Taiamã Ecological Station (TES) and its surrounding areas in northern Pantanal, Cáceres, Mato Grosso, Brazil. For effects including forest type, negative values = monospecific, positive = polyspecific. For effects including fire on site, negative = burned; positive = unburned. Thin line = 95% credible interval; thick line = 90% credible interval.


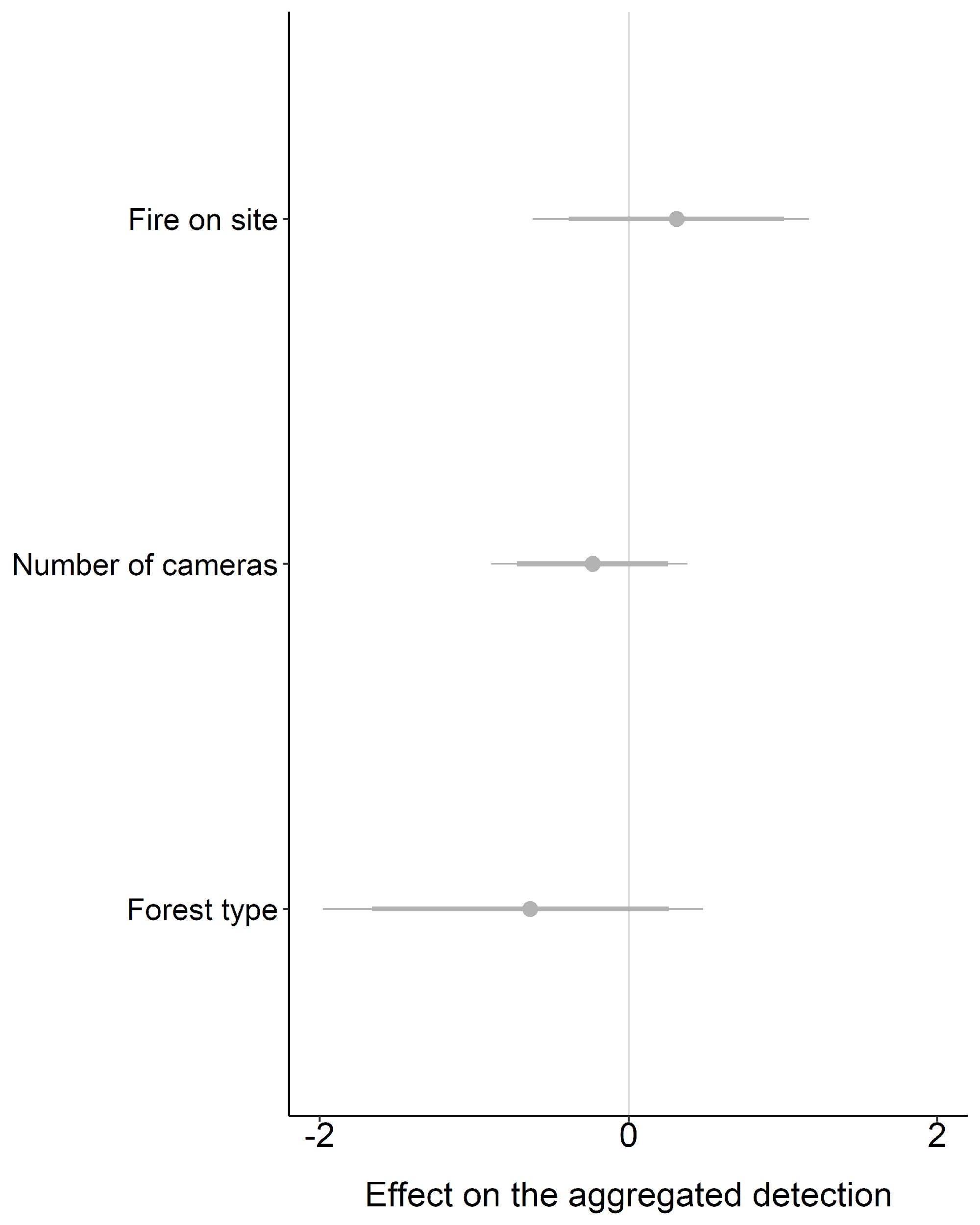


**Supplementary Figure 5.** Results of the Bayesian multi-species occupancy model, showing the magnitude and direction (Bayesian means ± credible intervals) for the posterior distributions of explanatory variables on aggregate mammal detection at the assemblage level in Taiamã Ecological Station (TES) and its surrounding areas in northern Pantanal, Cáceres, Mato Grosso, Brazil. For effects including forest type, negative values = monospecific, positive = polyspecific. For effects including fire on site, negative = burned; positive = unburned. Thin line = 95% credible interval; thick line = 90% credible interval.


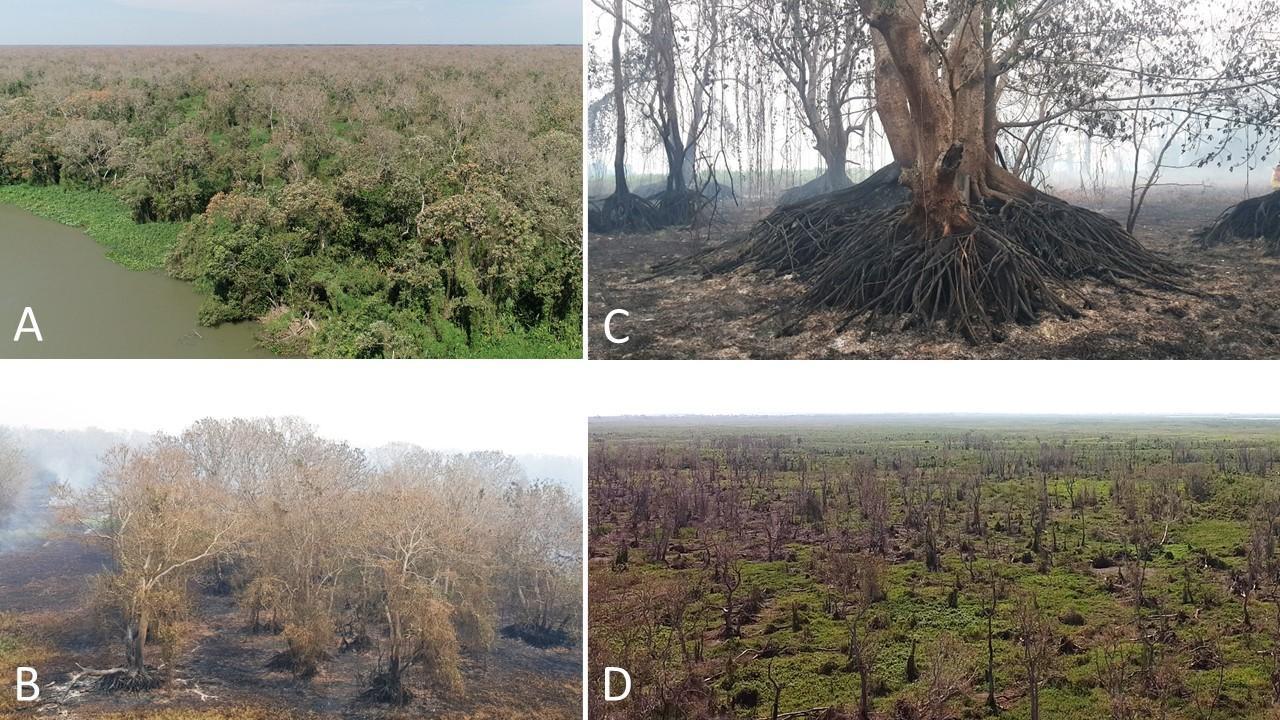


**Supplementary Figure 6.** Monospecific forests before (A – 2020-03-13) and after (B – 2020-10-01; C – 2020-09-13; D – 2021-08-19) the megafire event in September 2020 at Taiamã Ecological Station (TES) and its surrounding areas in northern Pantanal, Cáceres, Mato Grosso, Brazil.


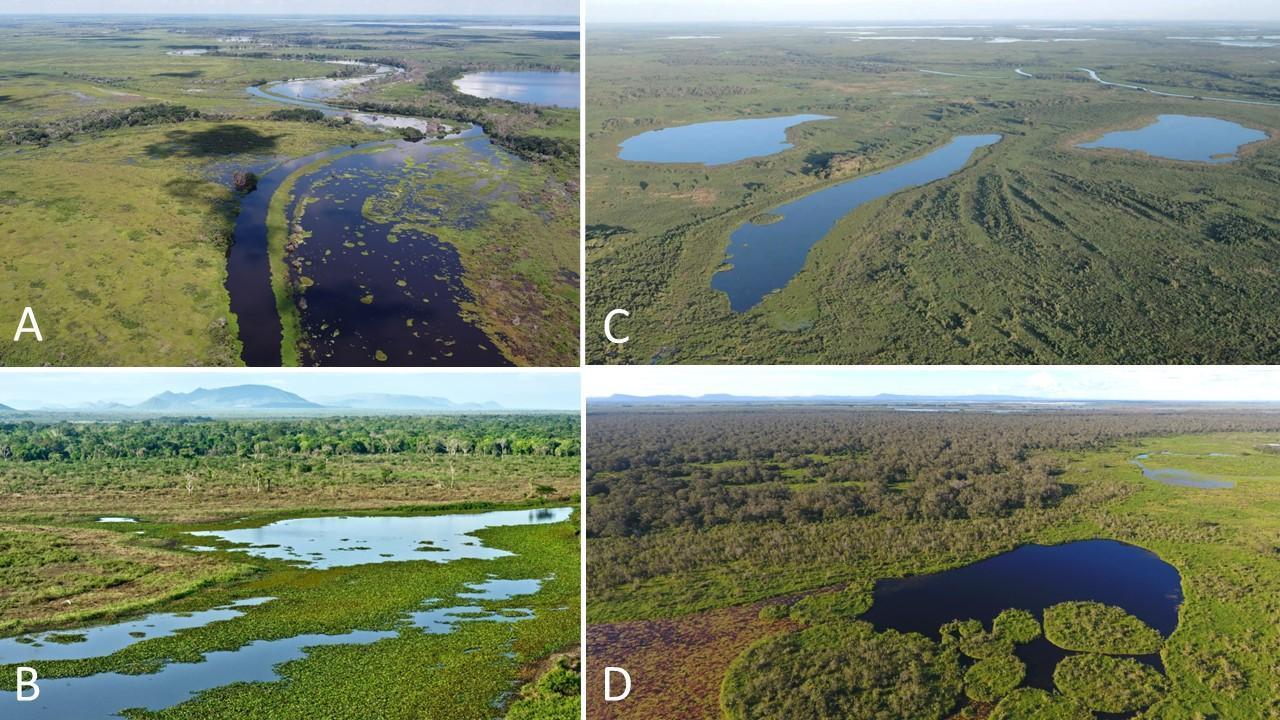


**Supplementary Figure 7.** Different aquatic habitats of the Taiamã Ecological Station (TES) and its surrounding areas in northern Pantanal, Cáceres, Mato Grosso, Brazil. (A) Period of floodplain and the Paraguay River. (B) A lagoon almost dry during the low water period. (C) Lagoons during the flood period. (D) Lagoons with floating vegetation.


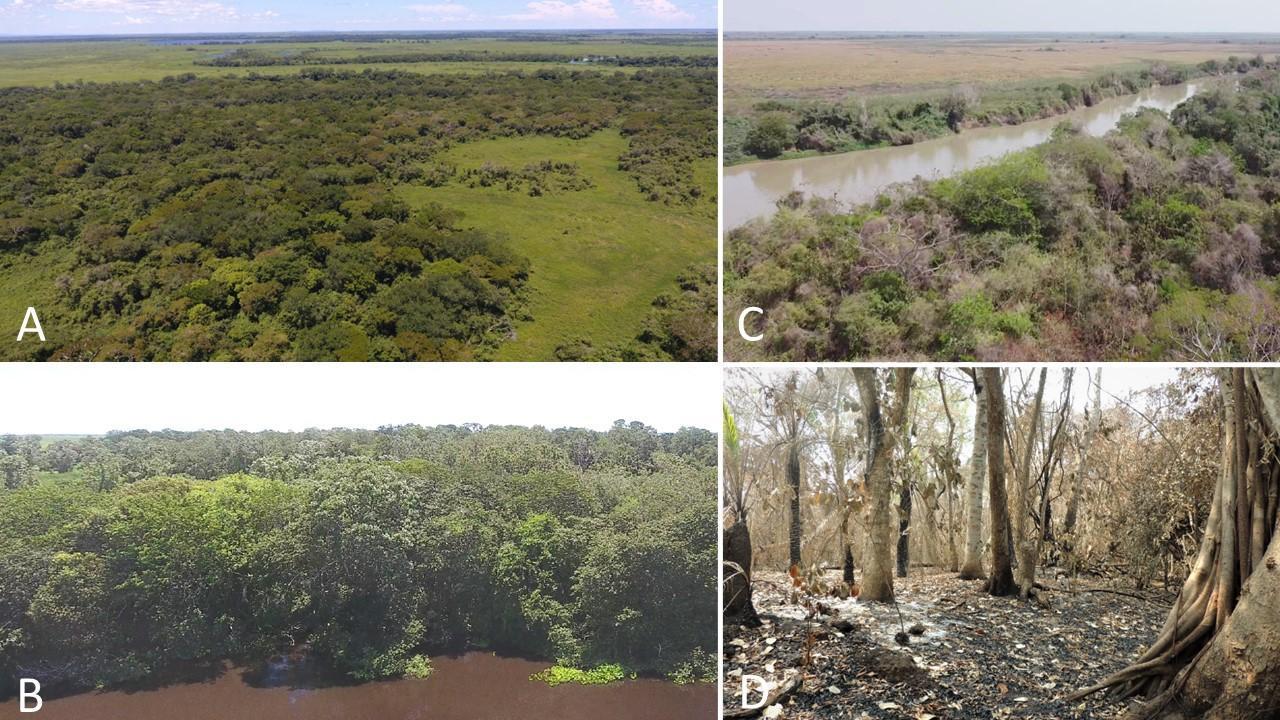


**Supplementary Figure 8.** Polyspecific forests before (A, B – 2018-12-13) and after (C – 2021-09-23; D – 2021-01-20) the megafire event in September 2020 at the Taiamã Ecological Station (TES) and its surrounding areas in northern Pantanal, Cáceres, Mato Grosso, Brazil.

**References**

1. Saranholi, B. H. *et al.* Comparing iDNA from mosquitoes and flies to survey mammals in a semi-controlled Neotropical area. *Mol. Ecol. Resour.* **23**, 1790–1799 (2023).

2. Rodgers, T. W. *et al.* Carrion fly-derived DNA metabarcoding is an effective tool for mammal surveys: Evidence from a known tropical mammal community. *Mol. Ecol. Resour.* **17**, e133–e145 (2017).

3. Kocher, A. *et al.* Evaluation of short mitochondrial metabarcodes for the identification of Amazonian mammals. *Methods Ecol. Evol.* **8**, 1276–1283 (2017).

4. Paglia, A. P. *et al.* Lista Anotada dos Mamíferos do Brasil 2^a^ Edição. *Occas. Pap. Conserv. Biol.* **6**, 76 (2012).

5. Magioli, M. *et al.* Land-use changes lead to functional loss of terrestrial mammals in a Neotropical rainforest. *Perspect. Ecol. Conserv.* **19**, 161–170 (2021).

6. MMA – Ministério do Meio Ambiente. Portaria MMA n^o^ 148, de 7 de junho de 2022: Altera os Anexos da Portaria n^o^ 443, de 17 de dezembro de 2014, da Portaria n^o^ 444, de 17 de dezembro de 2014, e da Portaria n^o^ 445, de 17 de dezembro de 2014, referentes à atualização da Lista Nacional de Espéc. *Diário Of. União* **108**, 74 (2022).

7. IUCN - International Union for Conservation of Nature and Natural Resources. The IUCN Red List of Threatened Species. Version 2023-1. https://www.iucnredlist.org/ (2023).

8. Lázari, P. R. D., Santos-Filho, M. dos, Canale, G. R. & Graipel, M. E. Flood-mediated use of habitat by large and midsized mammals in the Brazilian Pantanal. *Biota Neotropica* **13**, 70–75 (2013).

9. Eriksson, C. E. *et al.* Extensive aquatic subsidies lead to territorial breakdown and high density of an apex predator. *Ecology* **103**, e03543 (2022).
